# Career trajectories, transition rates, and birthdate distributions: the rocky road from youth to senior level in men's European football

**DOI:** 10.3389/fspor.2024.1420220

**Published:** 2024-07-17

**Authors:** Paolo Riccardo Brustio, Alexander B. T. McAuley, Alexandru Nicolae Ungureanu, Adam Leigh Kelly

**Affiliations:** ^1^ Department of Clinical and Biological Sciences, University of Turin, Turin, Italy; ^2^ Neuromuscular Function Research Group, School of Exercise & Sport Sciences, University of Turin, Turin, Italy; ^3^ Research for Athlete and Youth Sport Development (RAYSD) Lab, Centre for Life and Sport Sciences (CLaSS), Faculty of Health, Education and Life Sciences, Birmingham City University, Birmingham, United Kingdom

**Keywords:** transition rate, identification and selection processes, RAE, underdog hypothesis, soccer, talent development, athlete development

## Abstract

This study aimed to assess youth-to-senior transition rates, quantify the magnitude of relative age effect (RAEs), and evaluate how RAEs affect these transitions in 9,527 men's national football players of England, France, Germany, Italy, and Spain. Regardless of national team, only −15%, 25%, and 40% of U17, U19, and U21 players successfully transitioned to the senior team, respectively, whilst −14%–24% progressed to senior level without being selected during youth. Data suggested a skewed birthdate distribution favouring relatively older players at U17, U19, and U21 levels across all countries, whereas RAEs were also present in England, Italy, and Spain at senior level. Youth-to-senior transition rates were modulated by birthdate at U17 and U19, whereby Q4 players were −2 and 1.5 times more likely to successfully transition at senior level than Q1 players, respectively. Selection at youth international level does not guarantee selection at senior level, but does make it more likely. Moreover, relatively younger athletes are disadvantaged in youth categories, although are more likely to transition to senior level once they have entered the pathway.

## Introduction

To identify youth athletes with the potential of ascending to the higher echelons of senior competition more efficiently, sport's governing bodies and federations have implemented systematic recruitment strategies (1). Athlete development and ultimately achieving expertise in sport at adulthood, however, is a dynamic, highly contextual, and multifactorial process that is difficult to navigate (2, 3). For instance, current performance and future prospects in specific sport contexts can be influenced by performer (e.g., anthropometric, genetic, physiological, and psychological factors), task (e.g., deliberate practice and play, specialisation and sampling), and environmental (e.g., relative age, birthplace, cultural influences, and socioeconomic effects) constraints (4–9). Nevertheless, it is still unclear how and to what extent an athlete's developmental trajectory is shaped by the interaction between these constraints across sports (4, 10–12).

This ambiguity makes the accurate selection of prospective high performing athletes extremely challenging, and is further confounded by the weak relationship that exists between early and future success in sport (2, 13). More specifically, being a high performer at youth levels does not guarantee that the athlete will also be a high performer at senior level. Many prospective and retrospective studies have reported similar results across different sporting contexts, whereby approximately 20% of senior international athletes also performed at the highest level during their youth (14, 15). These findings were reinforced by a recent review (16), which showed that 82% of international-level seniors had not reached youth international level, suggesting that successful youths and seniors are largely two disparate populations.

Identification and selection complexity exacerbate in team sports such as football (i.e., soccer), likely due to the added positional dimensions and the compensatory nature of athletic profiles, which makes it even more difficult to define “talent” or appraise “elite” performance (17–19). Combined with the selectors' cognitive biases when assessing the potential of athletes [see (20)], this reduces the potential accuracy and reliability of selection decisions, especially at younger ages (21). The predictive utility and validity of early identification processes in facilitating successful youth-to-senior transitions were weak in footballers across Europe [e.g., (1, 22–24)]. These studies indicate that being a high performer during childhood and early adolescence or selected for a youth international roster is a poor predictor to obtain a professional contract and overall success at senior level. An evaluation based on current performance rather than their future developmental potential may partially explain the low success during youth-to-senior transitions. Such a reliance on static, objective measurements at one-off timepoints and subjective preferences based on gut-instinct, as well as the emphasis of youth sport organisations towards short-term success, undoubtedly compromises long-term athlete development and the ability to achieve expertise (2, 13).

An additional consequence of the currently implemented practices, however, is that they create contexts whereby biases such as relative age effects (RAEs) can influence identification and selection processes (1, 11, 12, 25). RAEs are a well-known phenomenon in football that reflects the (dis)advantages generated by the interaction between chronological age and an annual cut-off criterion, which is commonly used to group youth players of similar developmental stages together. Consequently, however, there can be up to twelve months difference in the chronological age of players in the same annual age-group, or even twenty-four months in the case of biennial age groups. Being relatively older (i.e., being born near the start of a cut-off date) means these players will generally benefit from increased anthropometric, physiological, and psychosocial development to produce higher performance than their relatively younger peers (i.e., born near the end of a cut-off date) (8, 26, 27). As a result, more relatively older players appear to be (un)consciously selected by recruiters (e.g., coaches, scouts) compared to relatively younger players at youth level (1, 6, 28–30). The valuable developmental opportunities accompanying early selection (e.g., greater access to coaching, competition, facilities, specialist support) are afforded to these players, which may further confound identification and selection processes and ultimately youth-to-senior transitions.

RAEs in football depend on contextual factors, such as age group, competitive playing level, gender, playing position, and sociocultural context (i.e., attraction level, country, depth of competition, historical moment) (1, 6, 28–30). Of particular relevance is that RAEs at senior level are more complex than at youth level. Whilst some research found a residual bias (i.e., knock-on effects) whereby the overrepresentation of relatively older players during youth continues into senior levels [e.g., (30–32)], other studies found reversal effects [e.g., (6, 25, 30, 33, 34)]. Reversal effects may be explained by the comparatively greater challenge experienced by relatively younger players compared to their relatively older peers during early development (i.e., the “underdog hypothesis”) (35). These experiences may improve psychological, social, technical, and tactical skills that become more evident at older chronological ages when being relatively older is less advantageous (35).

The mechanisms underpinning the youth-to-senior transition rate in football remain unclear. Research on RAEs predominately examined high performing European clubs or international competitions, whereas limited evidence exists on the national systems of countries and across different playing positions (1, 25). Moreover, studies in football typically investigate the phenomenon using a cross-sectional approach focused on one point in time, generally at youth levels, without considering the players' career at senior levels, leading to a lack of knowledge regarding the relationship between birthdates and the likelihood of successfully transitioning from youth to senior levels (29, 36). As England, France, Germany, Italy, and Spain are among the most influential footballing nations in both Europe and the world, in terms of historical success and impact on the development of the game, our study focused on these countries in order to highlight differences or common factors that influence player selection. Therefore, the aims of the present investigation were to: assess the rate of transitions from youth to senior level (Part I), quantify the prevalence and magnitude of RAEs across playing positions (Part II), and evaluate quartile youth-to-senior transition rate in the national teams of England, France, Germany, Italy, and Spain (Part III).

## Methods

Football player data, including birthdates, playing positions (i.e., goalkeepers, defenders, midfielders, forwards), and number of call-ups (regardless of whether players played or not) of youth (i.e., U17, U19, U21) and senior national teams of England, France, Italy, Germany, and Spain, were obtained from open-access online databases provided by Transfermarkt in September 2023 (https://www.transfermarkt.com). The selection of these specific countries was based on their representativeness at a European level and the availability of extensive data dating back to the year 2000, ensuring a valid and reliable analysis of trends over more than two decades. Specifically, the data consists of rosters for U17, U19, U21, and Senior categories from 2002 to 2022. A total of 9,527 players (U17 = 32.4%; U19 = 33.5%; U21 = 22.4%; Senior  = 11.7%) were included for analysing RAEs. To analyse the youth-to-senior transition rates, we considered a subsample of players born between 1985 and 1998 (both years included) after removing duplicates. Thus, only players eligible for selection to Senior teams (i.e., all players who were called up at least once to their respective senior team) were included in the study. Due to the inclusion criteria (i.e., players born between 1985 and 1998), all the athletes considered were at least 24-years-old (1, 25). This sample comprised 3,001 players with representation from England (18.3%), France (19.7%), Italy (21.3%), Germany (23.6%), and Spain (17.1%). Only players eligible for selection to Senior teams were included in the study (25). Informed consent was not required as the data was publicly available. The study was conducted in compliance with the Ethics Committee of the University of Torino (protocol number: 0635113).

## Procedure and statistical analysis

### Part I. Youth-to-senior transition rate

To obtain a broad view of the youth-to-senior transition rate, we first considered the U17, U19, and U21 age groups as separate age groups in this way, we considered the direct transition to from U17, U19, and U21 to Senior teams. Then, given the possibility of various transition patterns from youth to senior careers, the following combinations were used:

- OnlyU17, OnlyU19, OnlyU21: Players only selected for the U17 or U19 or U19 national team and subsequently selected to the Senior national team.
- OnlySenior: Players who were never called to any youth category but selected directly to the Senior national team.
- U17, 19&21: Players selected for all youth categories and subsequently selected to the Senior national team.
- U17&19, U17&21, U19&21: Players selected for the U17 and U19, for the U17 and U21 or for U19 and U21 national teams and subsequently selected to the Senior national team.

For all these combinations, binomial proportion confidence interval (90% CI) was calculated.

### Part II. Relative age effects

Players were divided into four quarters (i.e., Q1 = January–March; Q2 = April–June; Q3 = July–September; Q4 = October–December) according to the FIFA selection year (i.e., from January to December). The observed quartile distributions for each age cohort were then compared to the expected quartile distributions using chi-squared goodness-of-fit tests (χ^2^). Due to birth distribution differences in the nations considered we arbitrary used as expected quartile distributions the 25% for each quartile. Cramer's V was considered as effect sizes (φ_c_). The following thresholds was used: φ_c_ 0.06 trivial, 0.06 < φ_c _≤ 0.17 small, 0.17 < φ_c _< 0.29 medium, and φ_c _≥ 0.29 large. To compere the proportion of players in the Q1 and Q4 the odds ratios (ORs) and 95% confidence intervals (CIs) were calculated. The analyses were performed separately for each nation, age groups and players positions. In addition, to evaluate RAEs according to the level of competition, we considered the median of the number of call-ups in the respective of age group and nation. Therefore, we arbitrary defined a low performer a player with a number of call-ups ≤ of the median and a high performer a player with a number of call-ups > of the median.

### Part III. Quartile youth-to-senior transition rate

Binomial proportion confidence intervals (90% CI) were calculated to determine the proportion of players for each quartile (i.e., Q1, Q2, Q3, Q4) who could transition from U17, U19, and U21 to the Senior team. Furthermore, binary regressions with logit link were carried out to determine the impact of the birth quartile on transition rates. Due to the small number of players in each age group and quartile, all analyses involved merging the five national teams together.

## Results

### Part I. Youth-to-senior transition rate

**Figure 1:**
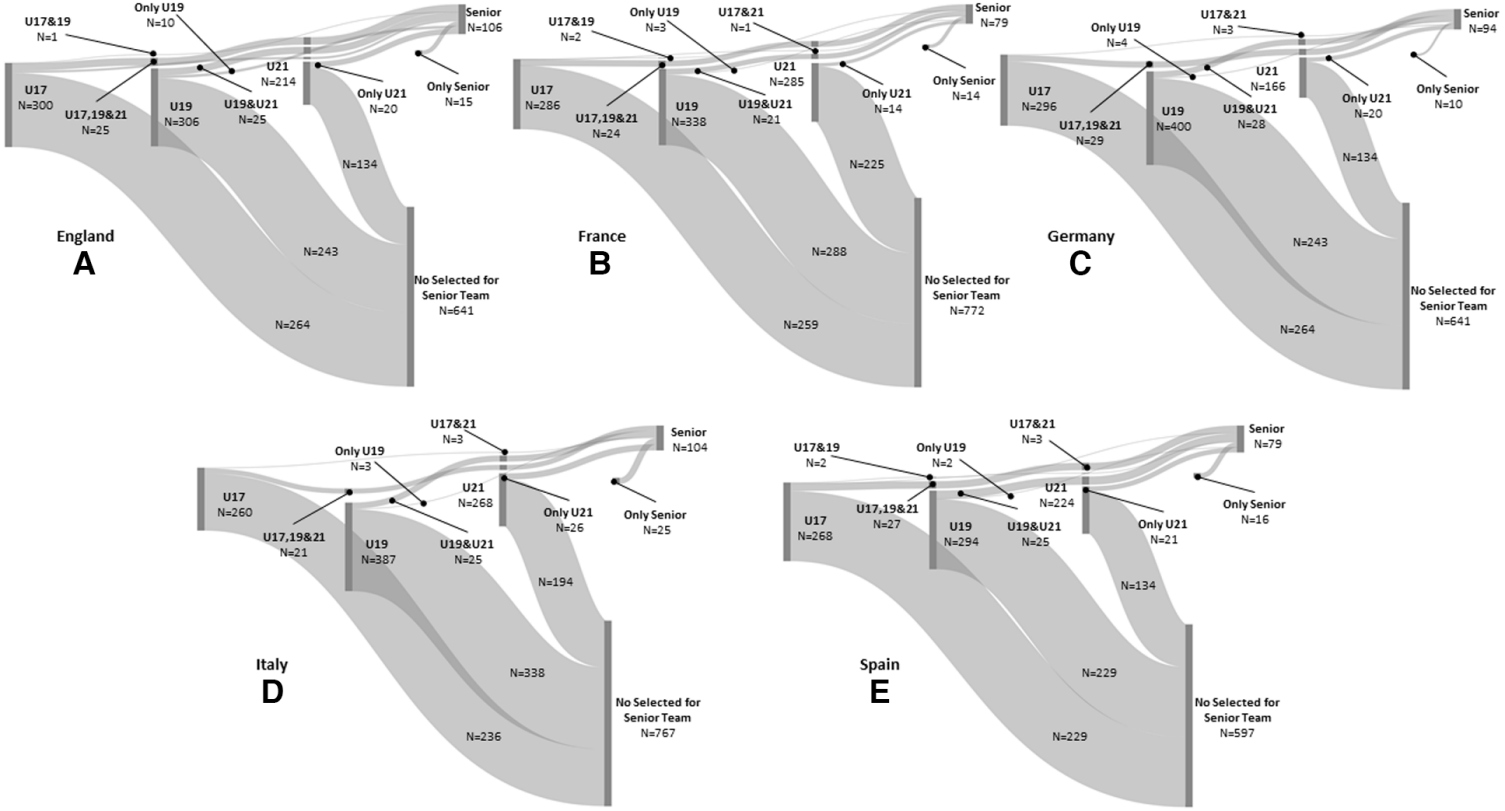
Overall visual inspection of the youth-to-senior transition rate in England (**A**), France (**B**), Germany (**C**), Italy (**D**), and Spain (**E**). The Sankey diagram provides the number of players able to reach the Senior national team from U17, U19, and U21 as well as the possible combinations. The figure also provides the number of players not selected for the Senior national team.

Fewer than 15% of U17 players progressed to the Senior team: England 12.0% (9.0, 15.5), France 9.4% (6.8, 12.8), Germany 9.8% (7.2, 12.9), Italy 9.2% (6.5, 12.7), and Spain 14.6% (11.1, 18.6). For U19 players, less than 25% progressed to the Senior team: England 20.6% (16.9, 24.8), France 14.8% (11.7, 18.4), Germany 12.7% (10.0, 15.8), Italy 13.2% (10.7, 16.1), and Spain 22.1% (18.2, 26.5). Finally, fewer than 40% of U21 players progressed to the Senior team: England 37.4% (31.9, 43.2), France 21.1% (17.1, 25.4), Germany 32.5% (27.6, 37.8), Italy 28.0% (23.5, 32.9), and Spain 38.0% (32.5, 43.6). Figure 1 provides an overall visual inspection of the youth-to-senior transition rate for each national team.

Generally, fewer than 15% of players selected for U17s were able to progress to U19s and U21s and then advance to their respective national Senior teams. National success rates were: England 9.0% (6.4, 12.2), France 8.4% (5.9, 11.6), Germany 8.8% (6.4, 11.9), Italy 8.1% (5.5, 11.4), and Spain 12.7% (9.5, 16.5). Moreover, less than 5% of U17 players were able to transition to the U19 or U21 teams and then to the Senior team, whilst no players were selected solely in the U17 team and then progressed to the Senior team. However, 0.7% to 3.3% (depending on the national teams) were selected solely for the U19 team before reaching the Senior team, whilst the transition rate to the Senior team increased to 8.3% in only U21 players. Finally, 14.2% to 24.0% of players reached the Senior team without youth national selection. See Table 1 for overall transition rates.

**Table 1:** Binomial proportion confidence interval [90% CI] of youth-to-senior transition rates in the different nations.

	England	France	Germany	Italy	Spain
U17 to senior	12.0 [9.0, 15.5]	9.4 [6.8, 12.8]	9.8 [7.2, 12.9]	9.2 [6.5, 12.7]	14.6 [11.1, 18.6]
U19 to senior	20.6 [16.9, 24.8]	14.8 [11.7, 18.4]	13.2 [10.7, 16.1]	12.7 [10.0, 15.8]	22.1 [18.2, 26.5]
U21 to senior	37.4 [31.9, 43.2]	21.1 [17.1, 25.4]	32.5 [27.6, 37.8]	28.0 [23.5, 32.9]	38.0 [32.5, 43.6]
Only U17	0	0	0	0	0
Only U19	3.3 [1.8, 5.5]	0.9 [0.2, 2.3]	0.9 [0.3, 2.0]	0.8 [0.2, 2.0]	0.7 [0.1, 2.1]
Only U21	9.4 [6.3, 13.3]	4.9 [3.0, 7.6]	8.1 [5.5, 11.6]	9.7 [6.9, 13.2]	9.4 [6.4, 13.2]
Only senior	14.2 [8.9, 21.0]	17.7 [11.0, 26.3]	10.6 [5.9, 17.4]	24.0 [17.3, 31.9]	15.2 [9.8, 22.2]
U17, 19&21 to senior	9.0 [6.4, 12.2]	8.4 [5.9, 11.6]	8.8 [6.4, 11.9]	8.1 [5.5, 11.4]	12.7 [9.5, 16.5]
U17&19 to Senior	0.3 [0.0, 1.6]	0.7 [0.1, 2.2]	0	0	0.8 [0.1, 2.3]
U17&21 to senior	2.7 [1.3, 4.8]	0.4 [0, 1.7]	0.9 [0.3, 2.4]	1.2 [0.3, 3.0]	1.1 [0.3, 2.9]
U19&21 to senior	8.3 [5.9, 11.4]	7.3 [5.0, 10.4]	8.5 [6.1, 11.5]	9.6 [6.8, 13.2]	10.1 [7.2, 13.6]

### Part II. Relative age effects

Table 2 summarises the relative age distribution and relative analysis of all players selected for the U17, U19, U21, and Senior national teams (i.e., playing positions individually and combined) in each nation. In addition, the table summarises the RAEs results considering the level of competition.

**Table 2:** Relative age outcomes.

	Age group	Playing position	N	Q1	Q2	Q3	Q4	χ^2^ (V,ES cat)	Q1 vs. Q4	N	Q1	Q2	Q3	Q4	χ2 (V, ES cat)	Q1 vs. Q4	N	Q1	Q2	Q3	Q4	χ^2^ (V, ES cat)	Q1 vs. Q4
		All competition level								Lower competition level							High competition level						
England	Under 17	All players	655	40.3	21.8	20.9	16.9	86.4*** (0.21, M)	2.4 (1.7, 3.2)	345	39.1	18.8	22.3	19.7	37.76*** (0.19, M)	2.0 (1.3, 3.0)	310	41.6	25.2	19.4	13.9	53.21*** (0.24, M)	3.0 (1.9, 4.8)
		Goalkeepers	72	41.7	18.1	26.4	13.9	13.0*** (0.25, M)	3.0 (1.4, 6.3)	48	35.4	22.9	27.1	14.6	4.33 (0.17, S)	2.4 (1.0, 6.2)	24	54.2	8.3	25.0	12.5	12.33** (0.41, L)	4.3 (1.2, 15.8)
		Defenders	208	40.4	23.1	19.7	16.8	27.88*** (0.21, M)	2.4 (1.5, 3.8)	111	38.7	20.7	18.0	22.5	11.54** (0.19, M)	1.7 (1.0, 3.1)	97	42.3	25.8	21.6	10.3	20.63*** (0.27, M)	4.1 (1.9, 8.8)
		Midfielders	209	41.1	20.6	20.1	18.2	29.48*** (0.22, M)	2.3 (1.5, 3.5)	104	39.4	14.4	25.0	21.2	13.92** (0.21, M)	1.9 (1.0, 3.4)	105	42.9	26.7	15.2	15.2	21.73*** (0.26, M)	2.8 (1.5, 5.4)
		Forwards	166	38.6	23.5	21.1	16.9	17.57*** (0.19, M)	2.3 (1.4, 3.7)	82	41.5	19.5	22.0	17.1	12.00** (0.22, M)	2.4 (1.2, 4.9)	84	35.7	27.4	20.2	16.7	7.14** (0.17, S)	2.1 (1.1, 4.3)
	Under 19	All players	561	35.8	21.6	22.1	20.5	35.9*** (0.15, S)	1.7 (1.3, 2.4)	303	32.7	22.1	22.1	23.1	9.57* (0.10, S)	1.4 (0.9, 2.2)	258	39.5	20.9	22.1	17.4	30.06*** (0.20, M)	2.3 (1.4, 3.7)
		Goalkeepers	68	35.3	20.6	22.1	22.1	3.88 (0.14, S)	1.6 (0.8, 3.2)	52	28.8	21.2	23.1	26.9	0.77 (0.07, S)	1.1 (0.5, 2.4)	16	56.3	18.8	18.8	6.3	4.00* (0.29, M)	9.0 (1.1, 72.9)
		Defenders	184	37.0	18.5	25.0	19.6	15.83*** (0.17, S)	1.9 (1.2, 3.0)	90	33.3	21.1	25.6	20.0	3.91 (0.12, S)	1.7 (0.9, 3.3)	94	40.4	16.0	24.5	19.1	13.08** (0.22, M)	2.1 (1.1, 4.0)
		Midfielders	166	33.1	26.5	19.9	20.5	7.57*** (0.12, S)	1.6 (1.0, 2.6)	90	30.0	23.3	17.8	28.9	3.39 (0.11, S)	1.0 (0.6, 2.0)	76	36.8	30.3	22.4	10.5	11.68** (0.23, M)	3.5 (1.5, 8.2)
		Forwards	143	37.8	20.3	21.0	21.0	12.36*** (0.17, S)	1.8 (1.1, 3.0)	71	38.0	22.5	22.5	16.9	6.94 (0.18, M)	2.3 (1.1, 4.8)	72	37.5	18.1	19.4	25.0	6.78 (0.18, M)	1.5 (0.8, 3.0)
	Under 21	All players	376	32.7	19.9	21.5	25.8	31.2*** (0.17, S)	1.3 (0.9, 1.8)	192	34.9	16.7	20.3	28.1	15.29*** (0.16, S)	1.2 (0.7, 2.1)	184	30.4	23.4	22.8	23.4	2.91 (0.07, S)	1.3 (0.7, 2.3)
		Goalkeepers	43	30.2	23.3	14.0	32.6	3.55 (0.17, S)	0.9 (0.4, 2.1)	31	29.0	19.4	12.9	38.7	4.63 (0.22, M)	0.8 (0.3, 1.9)	12	33.3	33.3	16.7	16.7	1.33 (0.19, M)	2.0 (0.4, 11.4)
		Defenders	140	35.0	17.1	25.0	22.9	9.31* (0.15, S)	1.5 (0.9, 2.6)	73	34.2	15.1	21.9	28.8	6.17 (0.17, S)	1.2 (0.6, 2.4)	67	35.8	19.4	28.4	16.4	6.18 (0.18, M)	2.2 (1.0, 4.9)
		Midfielders	104	29.8	26.0	21.2	23.1	1.77 (0.08, S)	1.3 (0.7, 2.3)	47	36.2	19.1	23.4	21.3	3.25 (0.15, S)	1.7 (0.7, 4.1)	57	24.6	31.6	19.3	24.6	1.79 (0.10, S)	1.0 (0.4, 2.3)
		Forwards	89	33.7	15.7	20.2	30.3	7.68 (0.17, S)	1.1 (0.6, 2.0)	41	39.0	14.6	19.5	26.8	5.70 (0.22, M)	1.5 (0.6, 3.4)	48	29.2	16.7	20.8	33.3	3.33 (0.15, S)	0.9 (0.4, 2.0)
	Senior	All players	219	31.1	17.8	21.0	30.1	18.40*** (0.17, S)	1.0 (0.6, 1.7)	112	34.8	17	18.8	29.5	9.86** (0.17, S)	1.2 (0.6, 2.4)	107	27.1	18.7	23.4	30.8	3.44** (0.10, S)	0.9 (0.4, 1.8)
		Goalkeepers	27	25.9	33.3	18.5	22.2	1.29 (0.13, S)	1.2 (0.4, 3.7)	20	25.0	40.0	10.0	25.0	3.60 (0.24, M)	1.0 (0.3, 3.9)	7	28.6	14.3	42.9	14.3	0.25 (0.11, S)	2.0 (0.2, 23.6)
		Defenders	76	30.3	14.5	23.7	31.6	5.58 (0.16, S)	1.0 (0.5, 1.9)	36	41.7	11.1	16.7	30.6	8.22 (0.28, M)	1.4 (0.5, 3.6)	40	20.0	17.5	30.0	32.5	2.60 (0.15, S)	0.6 (0.2, 1.8)
		Midfielders	55	36.4	18.2	14.5	30.9	6.93 (0.20, S)	1.2 (0.5, 2.5)	25	40.0	12.0	20.0	28.0	4.50 (0.24, M)	1.4 (0.5, 4.4)	30	33.3	23.3	10.0	33.3	4.25 (0.22, M)	1.0 (0.4, 2.8)
		Forwards	61	29.5	14.8	24.6	31.1	4.07 (0.15, S)	0.9 (0.4, 2.0)	31	29.0	12.9	25.8	32.3	2.63 (0.17, S)	0.9 (0.3, 2.6)	30	30.0	16.7	23.3	30.0	1.50 (0.13, S)	1.0 (0.3, 3.0)
France	Under 17	All players	647	49.0	25.5	17.5	8.0	237.90*** (0.35, L)	6.1 (4.2, 8.8)	335	50.7	23.0	17.9	8.4	132.82*** (0.36, L)	6.1 (3.7, 10.0)	312	47.1	28.2	17.0	7.7	107.72 (0.34, L)	6.1 (3.6, 10.4)
		Goalkeepers	74	54.1	20.3	20.3	5.4	36.74*** (0.41, L)	10.0 (3.5, 28.6)	53	50.9	24.5	18.9	5.7	23.46*** (0.38, L)	9.0 (2.6, 30.9)	21	61.9	9.5	23.8	4.8	17.80 (0.53, L)	13.0 (1.7, 101.8)
		Defenders	225	49.8	26.2	13.3	10.7	86.52*** (0.36, L)	4.7 (2.9, 7.6)	125	50.4	24.8	12.8	12.0	48.55*** (0.36, L)	4.2 (2.2, 80.0)	100	49.0	28.0	14.0	9.0	38.48 (0.36, L)	5.4 (2.5, 11.8)
		Midfielders	192	51.0	24.5	19.3	5.2	84.71*** (0.38, L)	9.8 (4.9, 19.5)	86	57.0	20.9	19.8	2.3	53.18*** (0.45, L)	24.5 (5.8, 104.3)	106	46.2	27.4	18.9	7.5	33.26 (0.32, L)	6.1 (2.7, 13.8)
		Forwards	156	42.9	28.2	19.9	9.0	38.41*** (0.29, M)	4.8 (2.6, 8.9)	71	43.7	21.1	23.9	11.3	15.50** (0.27, M)	3.9 (1.7, 9.0)	85	42.4	34.1	16.5	7.1	26.81 (0.32, L)	6.0 (2.4, 15.0)
	Under 19	All players	602	41.7	28.2	18.8	11.3	123.80*** (0.26, M)	3.7 (2.6, 5.2)	318	44.7	28.0	17.3	10.1	85.68 (0.30, L)	4.4 (2.7, 7.3)	284	38.4	28.5	20.4	12.7	41.38 (0.22, M)	3.0 (1.8, 5.0)
		Goalkeepers	60	45.0	25.0	18.3	11.7	14.93*** (0.29, M)	3.9 (1.6, 9.1)	40	45.0	25.0	20.0	10.0	10.4 (0.29, M)	4.5 (1.5, 14.0)	20	45.0	25.0	15.0	15.0	4.80 (0.28, M)	3.0 (0.8, 11.5)
		Defenders	199	42.7	30.2	15.6	11.6	48.30*** (0.28, M)	3.7 (2.2, 6.2)	114	44.7	30.7	15.8	8.8	34.55 (0.32, L)	5.1 (2.4, 10.8)	85	40.0	29.4	15.3	15.3	14.90 (0.24, M)	2.6 (1.3, 5.4)
		Midfielders	190	40.0	26.3	21.1	12.6	29.70 (0.23, M)	3.2 (1.9, 5.3)	89	44.9	24.7	18.0	12.4	21.86 (0.29, M)	3.6 (1.7, 7.7)	101	35.6	27.7	23.8	12.9	11.00 (0.19, M)	2.8 (1.4, 5.7)
		Forwards	153	41.2	29.4	20.3	9.2	34.18*** (0.27, M)	4.5 (2.4, 8.4)	75	44.1	29.3	17.3	9.3	20.26 (0.3, L)	4.7 (2.0, 11.4)	78	38.5	29.5	23.1	9.0	14.10 (0.25, M)	4.3 (1.8, 10.4)
	Under 21	All players	472	35.8	27.1	21.6	15.5	42.20*** (0.17, S)	2.3 (1.6, 3.4)	266	37.2	26.3	18.4	18.0	25.64*** (0.18, M)	2.1 (1.3, 3.3)	206	34.0	28.2	25.7	12.1	20.96*** (0.18, M)	2.8 (1.5, 5.1)
		Goalkeepers	52	40.4	26.9	13.5	19.2	8.46* (0.23, M)	2.1 (0.9, 4.7)	42	35.7	28.6	11.9	23.8	4.91 (0.20, M)	1.5 (0.6, 3.6)	10	60.0	0	20.0	20.0	0.67 (0.15, S)	3.0 (0.6, 15.6)
		Defenders	161	36.6	26.1	19.9	17.4	14.33** (0.17, S)	2.1 (1.3, 3.5)	96	42.7	24.0	14.6	18.8	17.75*** (0.25, M)	2.3 (1.2, 4.5)	65	27.7	29.2	27.7	15.4	3.31 (0.13, S)	1.8 (0.8, 4.3)
		Midfielders	136	33.8	25.0	26.5	14.7	10.12* (0.16, S)	2.3 (1.3, 4.1)	66	31.8	25.8	22.7	19.7	2.12 (0.10, S)	1.6 (0.7, 3.6)	70	35.7	24.3	30.0	10.0	10.00* (0.22, M)	3.6 (1.4, 9.0)
		Forwards	123	35.0	30.9	22.0	12.2	15.00** (0.20, M)	2.9 (1.5 5.4)	62	35.5	29.0	24.2	11.3	7.63 (0.20, M)	3.1 (1.2, 8.0)	61	34.4	32.8	19.7	13.1	7.93* (0.21, M)	2.6 (1.1, 6.5)
	Senior	All players	189	26.5	21.7	29.6	22.2	3.20 (0.08, S)	1.2 (0.7, 2.1)	95	26.3	24.2	29.5	20.0	1.79 (0.08, S)	1.1 (0.5, 2.4)	94	26.6	19.1	29.8	24.5	2.25 (0.09, S)	1.1 (0.5, 2.4)
		Goalkeepers	14	21.4	28.6	28.6	21.4	0.25 (0.08, S)	1.0 (0.2, 5.2)	10	20.0	30.0	40.0	10.0	0.33 (0.10, S)	0.5 (0.0, 5.9)	4	25.0	25.0	0.0	50.0	0.00 (0, T)	0.5 (0.0, 5.9)
		Defenders	70	22.9	17.1	34.3	25.7	4.22 (0.14, S)	0.9 (0.4, 1.9)	40	20.0	22.5	32.5	25.0	1.40 (0.11, S)	1.0 (0.3, 3.1)	30	26.7	10	36.7	26.7	4.25 (0.22, M)	1.0 (0.3, 3.1)
		Midfielders	62	37.1	19.4	24.2	19.4	5.13 (0.17, S)	1.9 (0.9, 4.3)	26	46.2	19.2	19.2	15.4	6.00 (0.28, M)	1.4 (0.5, 4.0)	36	30.6	19.4	27.8	22.2	1.11 (0.10, S)	1.4 (0.5, 4.0)
		Forwards	43	18.6	30.2	30.2	20.9	1.91 (0.12, S)	0.9 (0.3, 2.5)	19	15.8	31.6	31.6	21.1	1.40 (0.16, S)	1.0 (0.3, 3.9)	24	20.8	29.2	29.2	20.8	0.67 (0.10, S)	1.0 (0.3, 3.9)
Germany	Under 17	All players	695	50.2	28.5	14.4	6.9	327.80*** (0.40, L)	7.3 (5.0, 10.5)	368	48.6	28.5	14.9	7.9	142.13*** (0.36, L)	6.2 (3.8, 10.0)	327	52.0	28.4	13.8	5.8	161.01*** (0.41, L)	8.9 (5.1, 15.7)
		Goalkeepers	70	58.6	28.6	5.7	7.1	49.89** (0.49, L)	8.2 (3.2, 21.3)	46	60.9	23.9	8.7	6.5	33.50*** (0.49, L)	9.3 (2.7, 32.0)	24	54.2	37.5	0.0	8.3	18.33*** (0.50, L)	6.5 (1.4, 29.8)
		Defenders	227	49.8	29.5	13.7	7.0	98.12*** (0.38, L)	7.1 (4.0, 12.5)	120	48.3	27.5	13.3	10.8	42.60*** (0.34, L)	4.5 (2.3, 8.8)	107	51.4	31.8	14.0	2.8	57.52*** (0.42, L)	18.3 (5.5, 61.1)
		Midfielders	235	48.9	29.4	14.9	6.8	95.95*** (0.37, L)	7.2 (4.1, 12.7)	118	47.5	31.4	14.4	6.8	45.93*** (0.36, L)	7.0 (3.1, 15.6)	117	50.4	27.4	15.4	6.8	50.72*** (0.38, L)	7.4 (3.3, 16.5)
		Forwards	163	49.1	25.8	18.4	6.7	62.02*** (0.36, L)	7.3 (3.7, 14.2)	84	44.0	28.6	21.4	6.0	25.24*** (0.32, L)	7.4 (2.8, 19.8)	79	54.4	22.8	15.2	7.6	39.65*** (0.41, L)	7.2 (2.9, 17.8)
	Under 19	All players	833	43.2	26.4	19.6	10.8	346.4*** (0.37, L)	4.0 (2.9, 5.5)	435	41.1	28.5	19.3	11.0	86.89*** (0.26, M)	3.7 (2.5, 5.6)	398	45.5	24.1	19.8	10.6	103.82*** (0.29, M)	4.3 (2.8, 6.7)
		Goalkeepers	91	44.0	33.0	15.4	7.7	29.35*** (0.33, L)	5.7 (2.5, 13.2)	57	49.1	29.8	12.3	8.8	23.93*** (0.37, L)	5.6 (2.0, 15.3)	34	35.3	38.2	20.6	5.9	8.67* (0.29, M)	6.0 (1.3, 27.8)
		Defenders	276	42.8	26.1	21.0	10.1	61.04*** (0.27, M)	4.2 (2.6, 6.7)	149	37.6	28.2	23.5	10.7	22.46*** (0.22, M)	3.5 (1.8, 6.7)	127	48.8	23.6	18.1	9.4	43.28*** (0.34, L)	5.2 (2.6, 10.4)
		Midfielders	258	41.9	27.9	19.0	11.2	53.08*** (0.26, M)	3.7 (2.3, 5.9)	130	39.2	31.5	18.5	10.8	25.15*** (0.25, M)	3.6 (1.9, 7.2)	128	44.5	24.2	19.5	11.7	30.13*** (0.28, M)	3.8 (2.0, 7.3)
		Forwards	208	45.2	22.1	20.2	12.5	49.54*** (0.28, M)	3.6 (2.2, 5.9)	99	44.4	24.2	18.2	13.1	22.20*** (0.27, M)	3.4 (1.7, 6.8)	109	45.9	20.2	22.0	11.9	28.11*** (0.29, M)	3.8 (1.9, 7.7)
	Under 21	All players	436	36.2	26.8	22.2	14.7	42.00*** (0.18, M)	2.5 (1.7, 3.6)	219	31.1	28.3	24.7	16.0	11.25* (0.13, S)	1.9 (1.1, 3.4)	217	41.5	25.3	19.8	13.4	37.83*** (0.24, M)	3.1 (1.8, 5.5)
		Goalkeepers	45	26.7	33.3	22.2	17.8	2.45 (0.13, S)	1.5 (0.6, 3.8)	27	22.2	29.6	25.9	22.2	0.43 (0.07, S)	1.0 (0.3, 3.3)	18	33.3	38.9	16.7	11.1	3.60 (0.26, M)	3.0 (0.6, 15.6)
		Defenders	153	34.0	30.1	21.6	14.4	14.24** (0.18, M)	2.4 (1.4, 4.1)	71	28.2	31.0	22.5	18.3	2.72 (0.11, S)	1.5 (0.7, 3.4)	82	39.0	29.3	20.7	11.0	13.81** (0.24, M)	3.6 (1.5, 8.2)
		Midfielders	142	38.7	27.5	21.1	12.7	20.28*** (0.22, M)	3.1 (1.7, 5.5)	74	33.8	31.1	25.7	9.5	10.32* (0.22, M)	3.6 (1.4, 9.0)	68	44.1	23.5	16.2	16.2	14.24** (0.26, M)	2.7 (1.2, 6)
		Forwards	96	40.6	17.7	25.0	16.7	14.08** (0.22, M)	2.4 (1.3, 4.6)	47	36.2	19.1	25.5	19.1	3.58 (0.16, S)	1.9 (0.8, 4.6)	49	44.9	16.3	24.5	14.3	11.75** (0.28, M)	3.1 (1.2, 8)
	Senior	All players	200	30.0	24.0	26.5	19.5	5.70 (0.10, S)	1.5 (0.9, 2.7)	101	25.7	22.8	31.7	19.8	3.16 (0.10, S)	1.3 (0.6, 2.9)	99	34.3	25.3	21.2	19.2	5.32 (0.13, S)	1.8 (0.8, 3.9)
		Goalkeepers	18	27.8	38.9	22.2	11.1	2.80 (0.23, S)	2.5 (0.5, 13.5)	13	23.1	38.5	30.8	7.7	0.83 (0.15, S)	3.0 (0.3, 30.9)	5	40.0	40.0	0.0	20.0	2.50 (0.41, L)	2.0 (0.2, 23.6)
		Defenders	68	25.0	29.4	26.5	19.1	1.53 (0.09, S)	1.3 (0.6, 3.0)	37	21.6	29.7	32.4	16.2	2.56 (0.15, S)	1.3 (0.4, 4.4)	31	29.0	29.0	19.4	22.6	0.88 (0.10, S)	1.3 (0.4, 4.0)
		Midfielders	70	34.3	21.4	24.3	20.0	3.40 (0.13, S)	1.7 (0.8, 3.7)	32	28.1	15.6	31.3	25.0	1.75 (0.14, S)	1.1 (0.4, 3.4)	38	39.5	26.3	18.4	15.8	5.00 (0.21, M)	2.5 (0.8, 7.5)
		Forwards	44	31.8	13.6	31.8	22.7	4.00 (0.17, S)	1.4 (0.6, 3.5)	19	31.6	10.5	31.6	26.3	2.20 (0.20, M)	1.2 (0.3, 4.5)	25	32.0	16.0	32.0	20.0	2.17 (0.17, S)	1.6 (0.5, 5.6)
Italy	Under 17	All players	539	47.5	28.6	16.5	7.4	179.70*** (0.33, L)	6.4 (4.3, 9.5)	282	47.2	24.5	21.3	7.1	92.54*** (0.33, L)	6.7 (3.7, 11.8)	257	47.9	33.1	11.3	7.8	110.67*** (0.38, L)	6.2 (3.4, 11.0)
		Goalkeepers	57	47.4	24.6	21.1	7.0	19.50*** (0.34, L)	6.8 (2.3, 19.7)	39	43.6	23.1	28.2	5.1	11.50*** (0.31, L)	8.5 (1.9,38.0)	18	55.6	27.8	5.6	11.1	10.00* (0.43, L)	5.0 (1.1, 23.6)
		Defenders	179	45.3	33.0	12.3	9.5	62.33*** (0.34, L)	4.8 (2.7, 8.4)	87	41.4	33.3	16.1	9.2	22.95*** (0.3, L)	4.5 (2.0, 10.3)	92	48.9	32.6	8.7	9.8	41.48*** (0.39, L)	5.0 (2.3, 10.9)
		Midfielders	172	50.0	25.0	18.6	6.4	69.63*** (0.37, L)	7.8 (4.0, 15.2)	88	54.5	15.9	25.0	4.5	48.36*** (0.43, L)	12.0 (4.1, 34.9)	84	45.2	34.5	11.9	8.3	31.90*** (0.36, L)	5.4 (2.3, 12.9)
		Forwards	131	47.3	29.0	17.6	6.1	48.21*** (0.35, L)	7.8 (3.6, 16.7)	68	47.1	25.0	19.1	8.8	21.29*** (0.32, L)	5.3 (2.1, 13.5)	63	47.6	33.3	15.9	3.2	28.31*** (0.39, L)	15.0 (3.5, 64.9)
	Under 19	All players	670	38.8	26.9	21.6	12.7	113.30*** (0.24, M)	3.1 (2.2, 4.3)	378	37.0	27.2	22.2	13.5	43.64*** (0.20, M)	2.7 (1.8, 4.2)	292	41.1	26.4	20.9	11.6	53.29*** (0.25, M)	3.5 (2.1, 5.8)
		Goalkeepers	65	43.1	29.2	18.5	9.2	16.81*** (0.29, M)	4.7 (1.9, 11.6)	43	37.2	34.9	18.6	9.3	9.00* (0.26, M)	4.0 (1.3, 12.6)	22	54.5	18.2	18.2	9.1	10.00* (0.39, L)	6.0 (1.3, 27.8)
		Defenders	236	38.6	28.0	19.1	14.4	32.10*** (0.21, M)	2.7 (1.7, 4.2)	125	43.2	20.8	21.6	14.4	23.84*** (0.25, M)	3.0 (1.6, 5.6)	111	33.3	36.0	16.2	14.4	16.75*** (0.22, M)	2.3 (1.2, 4.5)
		Midfielders	206	43.2	24.3	19.9	12.6	41.73*** (0.26, M)	3.4 (2.1, 5.6)	118	39.8	25.4	18.6	16.1	15.80^***^ (0.21, M)	2.5 (1.3, 4.6)	88	47.7	22.7	21.6	8.0	29.00*** (0.33, L)	6.0 (2.5, 14.3)
		Forwards	163	31.9	27.6	28.8	11.7	16.02*** (0.18, M)	2.7 (1.5, 4.8)	92	25.0	34.8	29.3	10.9	11.57*** (0.2, M)	2.3 (1.0, 5.2)	71	40.8	18.3	28.2	12.7	12.83*** (0.25, M)	3.2 (1.4, 7.3)
	Under 21	All players	468	36.8	27.1	21.2	15.0	48.00*** (0.18, M)	2.5 (1.7, 3.6)	249	35.3	28.1	21.7	14.9	23.05*** (0.18, M)	2.4 (1.4, 4.0)	219	38.4	26.0	20.5	15.1	25.98 (0.2, M)	2.5 (1.5, 4.4)
		Goalkeepers	50	46.0	20.0	18.0	16.0	11.54** (0.28, M)	2.9 (1.2, 6.7)	36	41.7	22.2	19.4	16.7	5.56 (0.23, M)	2.5 (0.9, 6.9)	14	57.1	14.3	14.3	14.3	2.50 (0.24, M)	4.0 (0.8, 19.7)
		Defenders	179	30.7	30.7	21.2	17.3	9.89* (0.14, S)	1.8 (1.1, 3.0)	104	31.7	27.9	26.9	13.5	7.92* (0.16, S)	2.4 (1.1, 4.9)	75	29.3	34.7	13.3	22.7	7.53* (0.18, M)	1.3 (0.6, 2.7)
		Midfielders	129	41.1	28.7	18.6	11.6	25.59*** (0.26, M)	3.5 (1.9, 6.6)	66	37.9	31.8	18.2	12.1	10.94* (0.24, M)	3.1 (1.3, 7.6)	63	44.4	25.4	19.0	11.1	15.06*** (0.28, M)	4.0 (1.6, 10)
		Forwards	110	37.3	22.7	25.5	14.5	11.50** (0.19, M)	2.6 (1.4, 4.8)	43	34.9	27.9	16.3	20.9	3.36 (0.16, S)	1.7 (0.7, 4.1)	67	38.8	19.4	31.3	10.4	12.53** (0.25, M)	3.7 (1.5, 9.3)
	Senior	All players	273	31.1	26.7	24.5	17.6	53.60*** (0.26, S)	1.8 (1.0, 3.0)	139	30.9	30.2	21.6	17.3	7.40 (0.13, S)	1.8 (0.9, 3.6)	134	31.3	23.1	27.6	17.9	5.35 (0.12, S)	1.8 (0.9, 3.5)
		Goalkeepers	23	43.5	17.4	17.4	21.7	4.17 (0.25, S)	2.0 (0.6, 6.3)	16	31.3	18.8	25.0	25.0	0.00 (0.00, T)	1.3 (0.3, 5.2)	7	71.4	14.3	0.0	14.3	3.25 (0.39, L)	5.0 (0.5, 46.1)
		Defenders	91	29.7	29.7	18.7	22.0	3.35 (0.11, S)	1.4 (0.7, 2.7)	45	28.9	37.8	13.3	20.0	6.27 (0.22, M)	1.4 (0.5, 4.0)	46	30.4	21.7	23.9	23.9	0.83 (0.08, S)	1.3 (0.5, 3.4)
		Midfielders	79	30.4	31.6	26.6	11.4	8.15* (0.19, S)	2.7 (1.1, 6.3)	39	33.3	30.8	20.5	15.4	3.30 (0.17, S)	2.2 (0.7, 6.6)	40	27.5	32.5	32.5	7.5	6.8 (0.24, M)	3.7 (0.9, 14.8)
		Forwards	80	30.0	21.3	31.3	17.5	4.30 (0.13, S)	1.7 (0.8, 3.7)	39	30.8	25.6	30.8	12.8	3.30 (0.17, S)	2.4 (0.7, 7.9)	41	29.3	17.1	31.7	22.0	2.3 (0.14, S)	1.3 (0.5, 3.7)
Spain	Under 17	All players	548	50.0	29.0	14.2	6.8	217.50*** (0.36, L)	7.4 (4.9, 11.1)	297	53.2	30.0	11.1	5.7	165.01*** (0.43, L)	9.3 (5.1, 16.9)	251	46.2	27.9	17.9	8.0	79.86*** (0.33, L)	5.8 (3.2, 10.5)
		Goalkeepers	64	42.2	35.9	14.1	7.8	21.25*** (0.33, L)	5.4 (2.0, 14.4)	49	40.8	36.7	14.3	8.2	15.75** (0.33, L)	5.0 (1.6, 15.3)	15	46.7	33.3	13.3	6.7	5.13*** (0.34, L)	7.0 (0.8, 58.2)
		Defenders	193	52.3	30.6	14.0	3.1	106.98*** (0.43, L)	16.8 (7.2, 39.5)	104	57.7	26.9	12.5	2.9	71.46*** (0.48, L)	20.0 (6.0, 66.5)	89	46.1	34.8	15.7	3.4	39.41*** (0.38, L)	13.7 (4.1, 46.0)
		Midfielders	156	53.8	23.7	14.7	7.7	77.28*** (0.41, L)	7.0 (3.7, 13.3)	78	59.0	28.2	7.7	5.1	56.60*** (0.49, L)	11.5 (3.9, 33.5)	78	48.7	19.2	21.8	10.3	25.10*** (0.33, L)	4.8 (2.1, 10.8)
		Forwards	135	45.9	29.6	14.1	10.0	42.50*** (0.32, L)	4.4 (2.4, 8.2)	66	48.5	31.8	10.6	9.1	27.18*** (0.37, L)	5.3 (2.1, 13.5)	69	43.5	27.5	17.4	11.6	16.41*** (0.28, M)	3.8 (1.6, 8.7)
	Under 19	All players	527	41.7	26.9	20.3	11.0	102.20*** (0.25, M)	3.8 (2.6, 5.5)	285	38.6	28.4	20.7	12.3	43.11*** (0.22, M)	3.1 (1.9, 5.2)	242	45.5	25.2	19.8	9.5	65.80*** (0.30, L)	4.8 (2.7, 8.5)
		Goalkeepers	52	19.2	36.5	28.8	15.4	5.69 (0.19, M)	1.3 (0.5, 3.3)	35	20.0	37.1	28.6	14.3	4.11 (0.20, M)	1.4 (0.4, 4.6)	17	17.6	35.3	29.4	17.6	0.13 (0.05, T)	1.0 (0.2, 5.1)
		Defenders	194	50.0	28.4	13.9	7.7	81.22*** (0.37, L)	6.5 (3.6, 11.7)	103	46.6	29.1	14.6	9.7	33.73*** (0.33, L)	4.8 (2.3, 10.2)	91	53.8	27.5	13.2	5.5	48.91*** (0.42, L)	9.8 (3.7, 26)
		Midfielders	149	41.6	22.8	22.8	12.8	26.14*** (0.24, M)	3.3 (1.9, 5.7)	78	44.9	23.1	19.2	12.8	17.70*** (0.28, M)	3.5 (1.6, 7.6)	71	38.0	22.5	26.8	12.7	9.28* (0.21, M)	3.0 (1.3, 6.8)
		Forwards	132	38.6	25.8	23.5	12.1	18.73*** (0.22, M)	3.2 (1.7, 5.8)	69	29.0	29.0	27.5	14.5	4.18 (0.14, S)	2.0 (0.9, 4.6)	63	49.2	22.2	19	9.5	21.56*** (0.34, L)	5.2 (2, 13.1)
	Under 21	All players	385	39	24.9	20.5	15.6	54.20*** (0.22, M)	2.5 (1.7, 3.7)	214	39.3	20.6	21	19.2	23.15*** (0.19, M)	2.0 (1.2, 3.5)	171	38.6	30.4	19.9	11.1	29.47*** (0.24, M)	3.5 (1.8, 6.7)
		Goalkeepers	36	19.4	38.9	22.2	19.4	3.78 (0.19, S)	1.0 (0.3, 2.9)	21	23.8	33.3	28.6	14.3	1.80 (0.17, S)	1.7 (0.4, 7.3)	15	13.3	46.7	13.3	26.7	0.63 (0.12, S)	0.5 (0.1, 2.8)
		Defenders	142	49.3	19.0	19.7	12.0	46.17*** (0.33, L)	4.1 (2.3, 7.4)	84	47.6	16.7	17.9	17.9	22.95*** (0.30, L)	2.7 (1.3, 5.4)	58	51.7	22.4	22.4	3.4	26.80*** (0.39, L)	15.0 (3.4, 66)
		Midfielders	116	37.1	26.7	19.8	16.4	11.59** (0.18, M)	2.3 (1.2, 4.1)	56	39.3	19.6	21.4	19.6	6.14 (0.19, M)	2.0 (0.9, 4.5)	60	35.0	33.3	18.3	13.3	8.40* (0.22, M)	2.6 (1.1, 6.5)
		Forwards	91	33.0	26.4	22.0	18.7	4.13 (0.12, S)	1.8 (0.9, 3.4)	53	32.1	22.6	22.6	22.6	1.46 (0.10, S)	1.4 (0.6, 3.3)	38	34.2	31.6	21.1	13.2	4.20 (0.19, M)	2.6 (0.9, 7.8)
	Senior	All players	232	34.5	26.7	20.3	18.5	28.30*** (0.20, S)	1.9 (1.1, 3.2)	116	37.9	26.7	16.4	19.0	13.03*** (0.19, M)	2.0 (1.0, 4.1)	116	31.0	26.7	24.1	18.1	4.07 (0.11, S)	1.7 (0.8, 3.6)
		Goalkeepers	23	17.4	26.1	17.4	39.1	2.83 (0.20, S)	0.4 (0.1, 1.5)	16	18.8	25.0	18.8	37.5	0.25 (0.07, S)	0.5 (0.1, 2.2)	7	14.3	28.6	14.3	42.9	0.25 (0.11, S)	0.3 (0.0, 3.4)
		Defenders	77	41.6	24.7	18.2	15.6	12.79** (0.24, S)	2.7 (1.2, 5.8)	37	35.1	32.4	10.8	21.6	5.67 (0.23, M)	1.6 (0.6, 4.6)	40	47.5	17.5	25.0	10.0	12.60*** (0.32, L)	4.8 (1.4, 16.1)
		Midfielders	69	36.2	31.9	20.3	11.6	10.53* (0.23, S)	3.1 (1.3, 7.6)	34	50.0	26.5	17.6	5.9	13.56*** (0.36, L)	8.5 (1.8, 40.9)	35	22.9	37.1	22.9	17.1	3.00 (0.17, S)	1.3 (0.4, 4.4)
		Forwards	63	30.2	23.8	23.8	22.2	0.94 (0.07, S)	1.4 (0.6, 3.0)	29	37.9	20.7	20.7	20.7	2.71 (0.18, M)	1.8 (0.6, 5.8)	34	23.5	26.5	26.5	23.5	0.22 (0.05, T)	1.0 (0.3, 3.1)

Q1, ﬁrst quartile percentage; Q2, second quartile percentage; Q3, third quartile percentage; Q4, fourth quartile percentage; χ^2^, chi-square value; V, Cramer's V effect size; effect size category: T, trivial = V ≤ 0.06; S, small = 0.06 < V ≤ 0.17; M, medium = 0.17 < V < 0.29; L, large = V ≥ 0.29; OR, odds ratio and 95% conﬁdence intervals (95% CI); Q1 vs. Q4, ﬁrst versus the last quartile.

*p<0.05, **p<0.01, ***p<0.001.

Regardless of the country, RAEs were observed in all positions for U17, U19, and U21 players, although decreasing with age. In U17, the effect size was large (φ_c _= 0.40–0.35), U19 ranged from large-to-small (φ_c _= 0.37–0.15), and U21 was medium-to-small (φ_c _= 0.22–0.17). The odd to be selected in Q1 was greater than in Q4, with mean OR values of 5.9, 3.2, and 2.2 in U17, U19, and U21, respectively. At the Senior level, medium-to-small RAEs persisted in English, Italian, and Spanish teams (φ_c _= 0.26–0.17), but were absent in French and German teams.

Similar trends were observed when analysing the distributions by playing position. At U17 and U19 levels, RAEs were present in all positions with a large-to-medium effect size (φ_c _= 0.49–0.12). For U21 teams, the Italian and French selections presented birth-skewed distribution in all positions (φ_c _= 0.29–0.16), the German selections presented birth-skewed distribution in all positions except goalkeepers (φ_c _= 0.22–0.18), and the English and Spanish selections presented birth-skewed distribution only in defenders and defenders/midfielders, respectively (φ_c _= 0.33–0.15). At the Senior level, only midfielders in the Italian team and defenders/midfielders in the Spanish team showed a birth-skewed distribution. However, the overall mean indicated that players born in the first half of the year (%) were more frequently represented than those born in the second half (%).

The median number of calls required to identify the players called up was 4–5 for the U17s, 3–5 for the U19s, 5–7 for the U21s, and 7–14 for Senior teams. When considering high and low performers, RAEs were more pronounced, with a higher effect size for high performer players. High performers born in Q1 were more likely to be selected than those born in Q4, with mean ORs (merged for all nations) of 6.0, 3.6, and 2.6 at U17, U19, and U21, respectively. In low performers, the ORs were 6.1, 3.0, and 1.9 at U17, U19, and U21, respectively. As expected, the magnitude of RAEs decreased with age and is less pronounced in the Senior teams. Interestingly, a significant inverse RAE was observed for England in high performer players. See Supplementary File S1 for quartile distribution based on nation, playing positions, and competition level.

### Part III. Quartile youth-to-senior transition rate

**Figure 2:**
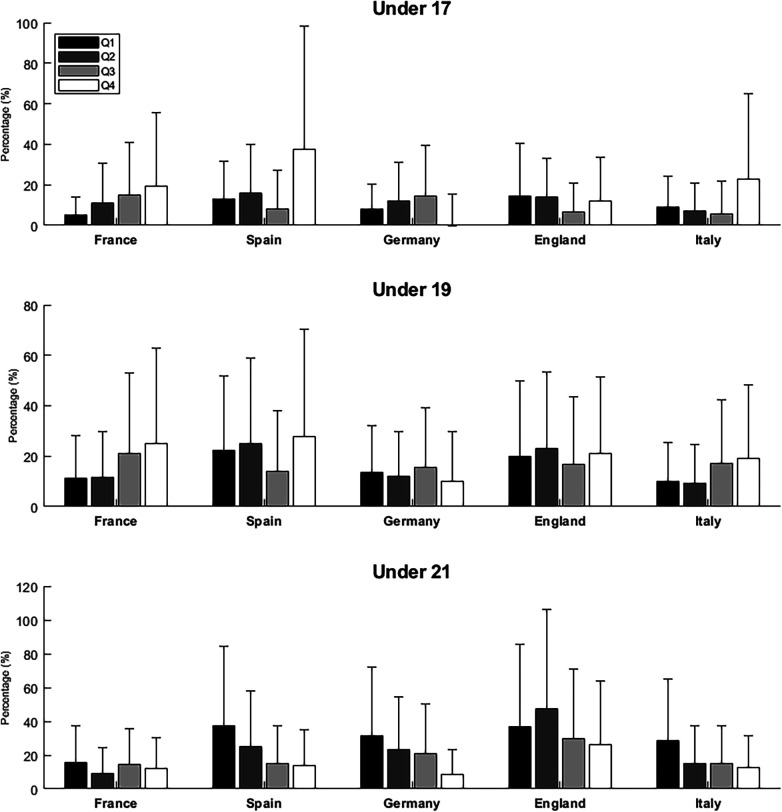
Figure present the frequency of successful birth quartile transition rate for players in U17, U19, and U21.

Figure 2 provides the transition rate by quartile for players selected in U17, U19, and U21. A higher percentage of players born in Q4 transitioned to Senior teams compared to Q1 players, especially in U17 and U19 teams (overall average: Q1 = 9.0 (7.2, 11.2) vs. Q4 = 16.4 (11.5, 22.5) and Q1 = 14.6 (12.4, 17.0) vs. Q4 = 20.0 (16.0, 24.6) for U17 and U19, respectively). For the U21s, the trend was similar (overall average: Q1 = 28.7 (25.3, 32.3) vs. Q4 = 33.5 (27.8, 37.6).

The Q4 players of U17 and U19 had 1.98 (1.15, 3.32) and 1.47 (1.00, 2.13) higher odds of transitioning to the Senior teams when compared to Q1 players, respectively. Contrastingly, in U21, the transition trends were similar for the quartile with no differences observed in logistic regression [e.g., OR =1.25 (0.87, 1.80) for Q1 vs. Q4]. See Supplementary File S2 for binary regressions with logit link.

## Discussion

Using five European national teams, this study aimed to assess the rate of transition from youth to senior level (Part I), evaluate the prevalence and magnitude of RAEs across different nations and playing positions (Part II), and assess the quartile transition rate from youth to senior level (Part III). Regardless of national team, −15%, less than 25%, and less than 40% of U17, U19, and U21 players, respectively, were successfully selected for their Senior team. Additionally, −14%–24% of players (depending on national team) were selected only at Senior level (Part I). Moreover, data suggested a skewed birthdate distribution favouring relatively older players at U17, U19, and U21 level (on average Q1 = 38.7% vs. Q4 = 15.2%), while RAEs were present depending on the national context at Senior level (Part II). RAEs were also prevalent in all player positions to some extent, most notably at U17 and U19 with medium/large effect sizes. Moreover, RAEs were pronounced at higher competition level (i.e., players who had been called up to the national youth team more often). Finally, the youth-senior transition rate is modulated by birthdate at U17 and U19, where Q4 players were −2 and 1.5 times more likely to transition to the Senior team than Q1 players, respectively (Part III).

Analysis of the youth-to-senior transition rate suggests that, irrespective of national context, players selected at youth national level are not necessarily successful at Senior level. Indeed, only −25% were able to successfully transition to Senior teams in the U17 and U19 categories. As players got older, however, around a third who were selected at U21 level successfully transitioned to their Senior team (from 21.1 to 38.0%). Moreover, selected later-born players had an increased likelihood of completing the transition (24, 30). These results are in line with previous studies in male (1) and female (25) footballers, and underline how transition rates are modulated by age group (i.e., an increasing likelihood of being selected in older age groups). For instance, Boccia et al. (1) revealed that less than 10% of U16 national team players successfully transition to Senior teams, while −40% of U21 players were eventually chosen for Senior teams.

In terms of national comparisons, when the transition rates for all youth categories were combined, England (23.3%) and Spain (24.9%) presented the highest rates followed by Germany (18.5%), Italy (16.6%), and France (15.1%). Overall, these results suggest that, independent of national context, selection in national youth teams cannot be considered a key factor for future selection at the Senior level. It is interesting to note that the transition rates drop significantly when considering the direct transition from a youth category (i.e., U17, U19, or U21) to the Senior category (i.e., less than 9% of players). These findings highlight that being selected in at least two youth national teams may increase the chances of being selected for the Senior team (1). Conversely, around a quarter of the senior players were not selected at youth level. Put simply, youth categories are likely underachieving as there is no clear pathway, especially considering that the majority of players in the youth categories are not selected again. It should also be noted that, at the youth levels, there tends to be a lot of selection initially followed by a significant amount of exclusion, further highlighting the complex and nuanced trajectories from youth to senior levels. Taken together, the data shows a high turnover of youth players and low likelihood of being selected for Senior teams (37), which is likely due to repeated (de)selection procedures throughout childhood and adolescence rather than early selection and long-term continuous development (23).

RAE analysis showed that, regardless of the cultural context, there were consistent asymmetries in quartile distribution within U17, U19, and U21 categories, whereby relatively older players overrepresented compared to relatively younger equivalents (−36% in Q1 vs. −19% in Q4). Specifically, players born in Q1 were −6, −3, and −2 times more likely to be selected than those born in Q4 in U17, U19, and U21, respectively. On the other hand, however, although the magnitude of RAEs were small at Senior levels, they were still present in English, Italian, and Spanish teams (φ_c _= 0.17–0.26). These results confirm that age modulates the magnitude of RAEs, and that this decreases with increasing age (38). Interestingly, in some senior national contexts (i.e., England, Italy, Spain), our data showed a residual bias indicating knock-on effects. These findings highlight the ongoing over-representation of relatively older players from youth to senior levels, highlighting the complex dynamics of age-related advantages in football and suggesting that the impact of RAEs may evolve and manifest differently depending on the national context.

The analysis of the playing position offers additional information regarding the mechanisms of RAEs in European football rosters. RAEs were also prevalent in all player positions, most notably at U17 and U19 with medium/large effect sizes. Regardless of nationality, goalkeepers born in Q1 were on average −7 and 4 times more likely to be selected than those born in Q4 at U17 and U19, respectively. For other playing positions, the magnitude of the RAE changes in relation to the national context. For example, at U17 level, RAEs were more prevalent among midfielders in France, defenders in Spain, midfielders and forwards in Germany and Italy, whilst being similar in all player positions in England. This data may suggest how the magnitude of RAEs is higher in players position where more developed physical qualities may provide a competitive advantage. From the U21 level onwards, RAEs were only observed for some player positions, depending on the socio-cultural context. For example, the lowest OR was observed in England and Spain, suggesting that these two countries were able to mitigate RAEs through a more balanced selection policy. The results suggest that the impact of chronological age in the playing position varies according to the socio-cultural context (29, 32, 38). These differences may be based on the country-specific differences and playing styles. However, these are only speculations, and given that the nations included are among the most influential footballing nations in Europe, both in terms of historical success and impact on the development of the game, this aspect should be addressed in future studies.

In accordance with previous studies (26, 38), data suggested that RAEs were more pronounced at higher competition level (i.e., players who had been called up to the national youth team more often were generally relatively older). Additionally, players born in Q1 were −6, 4, and 3 times more likely to be selected than those born in Q4 at high competition level, while players born in Q1 were −6, 3, and 2 at U17, U19, and U21 for the low competition level, respectively. Overall, data confirms that country, playing position, and competition level influence the extent of RAEs, which is likely varies according the national team philosophy and subsequent playing style (39).

Focusing on the quartile transition, the results show how players born in Q4 had an advantage in the youth-to-senior transition, highlighting a reversal of the relative age advantage at senior level and consequently confirming the “underdog hypothesis” (35). Q4 players were more likely to make the transition from U17 or U19 to senior level than Q1 players (i.e., −2 and 1.5 times more likely than those born in Q4, respectively). At U21 level, a trend was also observed, although it was not statistically significant (i.e., 1.25 times more likely). These inverse effects can be explained by the “underdog hypothesis”, whereby relatively younger players face a greater challenge in comparison to their relatively older counterparts during early development. These experiences may enhance a higher degree of psychological resilience and toughness (40), as well as higher skill proficiency (i.e., social, technical, tactical skills) that allows relatively young players to overcome initial birthdate disadvantages and increase their chances of making senior level (35, 41). However, it is important to remember these suggestions remain hypothetical and the exact mechanisms contributing to these trends have yet to be determined.

## Limitations

This study did not explore factors influencing player development trajectories, such as injuries, coaching quality, socioeconomic background, or motivation. We have defined inclusion in the Senior category based on having at least one call-up. Consequently, this criterion may introduce a selection bias when interpreting the results. Moreover, our study describes the youth-to-senior transition rate, considering national call-ups, but did not examine youth development structures. Whilst this approach was taken for simplicity, it did not allow us to investigate the mechanisms that underpin the selection process. There is scope for further investigation, including female contexts, career trajectories across the lifespan, and long-term outcomes of players who were not selected for national teams but may still excel in high-level club competitions.

## Conclusion

Our results show that being a high-performing youth international player is not a sufficient proxy for reaching senior national team level. In addition, the data suggest that, although RAEs can influence selection, especially in youth categories, individuals born further from the cut-off date have a higher likelihood of successfully transitioning through to senior teams once selected into the national team pathway.

## Data Availability

Publicly available datasets were analyzed in this study. This data can be found here: https://www.transfermarkt.com/.

## Supplementary material

Supplementary File S1 Birth quartile percentage distributions presented individually for all playing positions (i.e. goalkeepers, defenders, midfielders and forwards) for U17, U19, U21 and senior for each nation.
