# Supplementary material for: Career trajectories, transition rates, and birthdate distributions: the rocky road from youth to senior level in men's European football
Source: Front Sports Act Living. 2024 Jul 17;6:1420220. doi: 10.3389/fspor.2024.1420220 (PMC11288826; doi:10.3389/fspor.2024.1420220)
Supplement: Supplementary File S1 — Birth quartile percentage distributions presented individually for all playing positions (i.e. goalkeepers, defenders, midfielders and forwards) for U17, U19, U21 and senior for each nation. [file Datasheet1.docx]

Supplementary 1


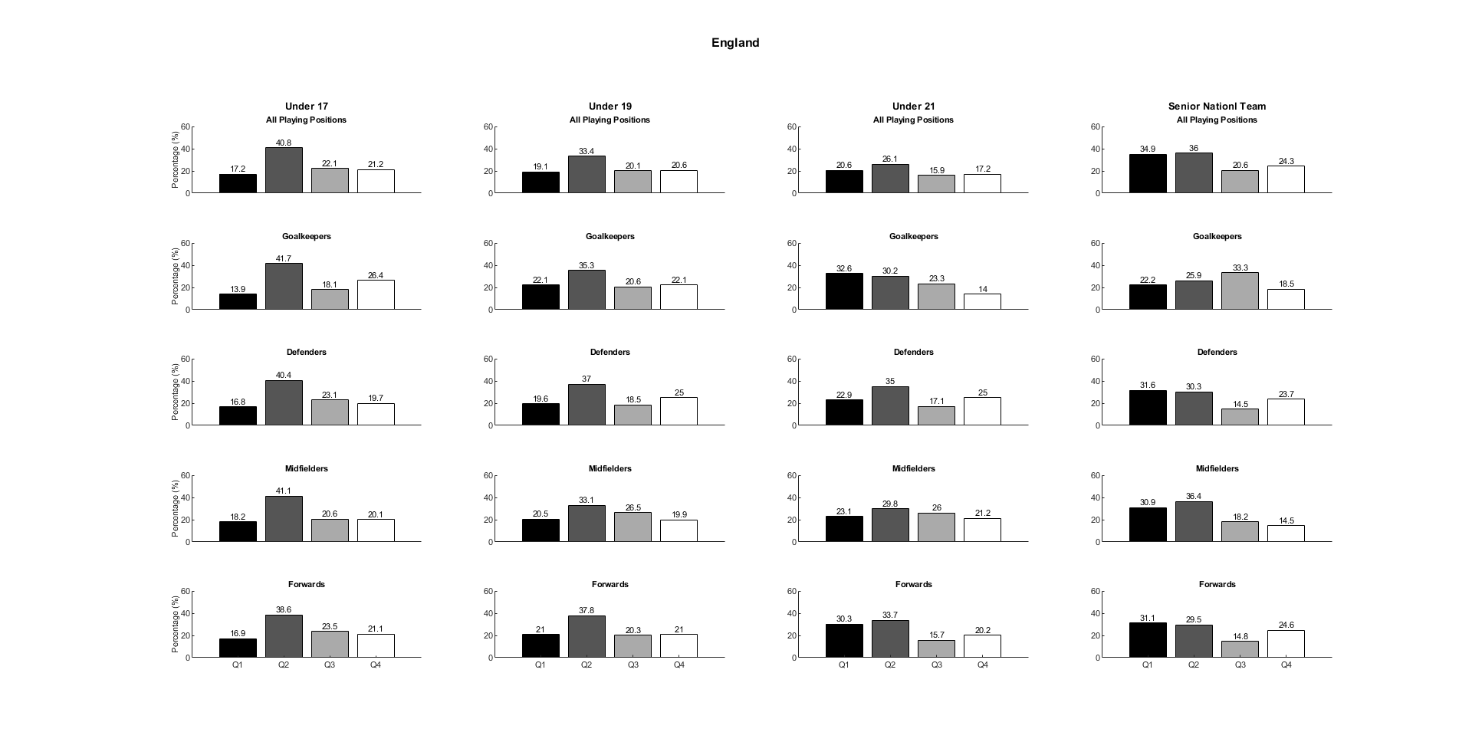


Legend: Birth quartile percentage distributions presented individually for all playing positions (i.e. goalkeepers, defenders, midfielders and forwards) for U17, U19, U21 and senior English players.


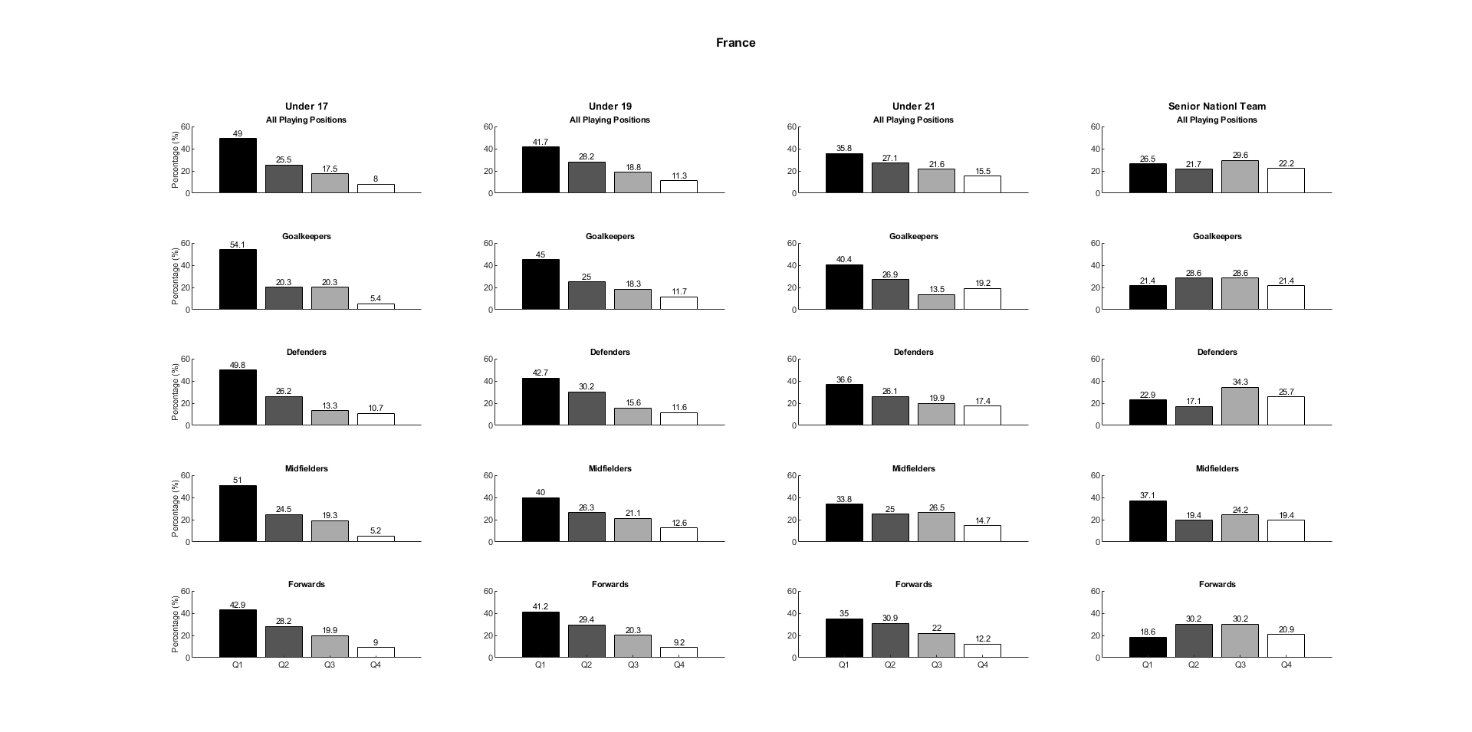
 Legend: Birth quartile percentage distributions presented individually for all playing positions (i.e. goalkeepers, defenders, midfielders and forwards) for U17, U19, U21 and senior French players.


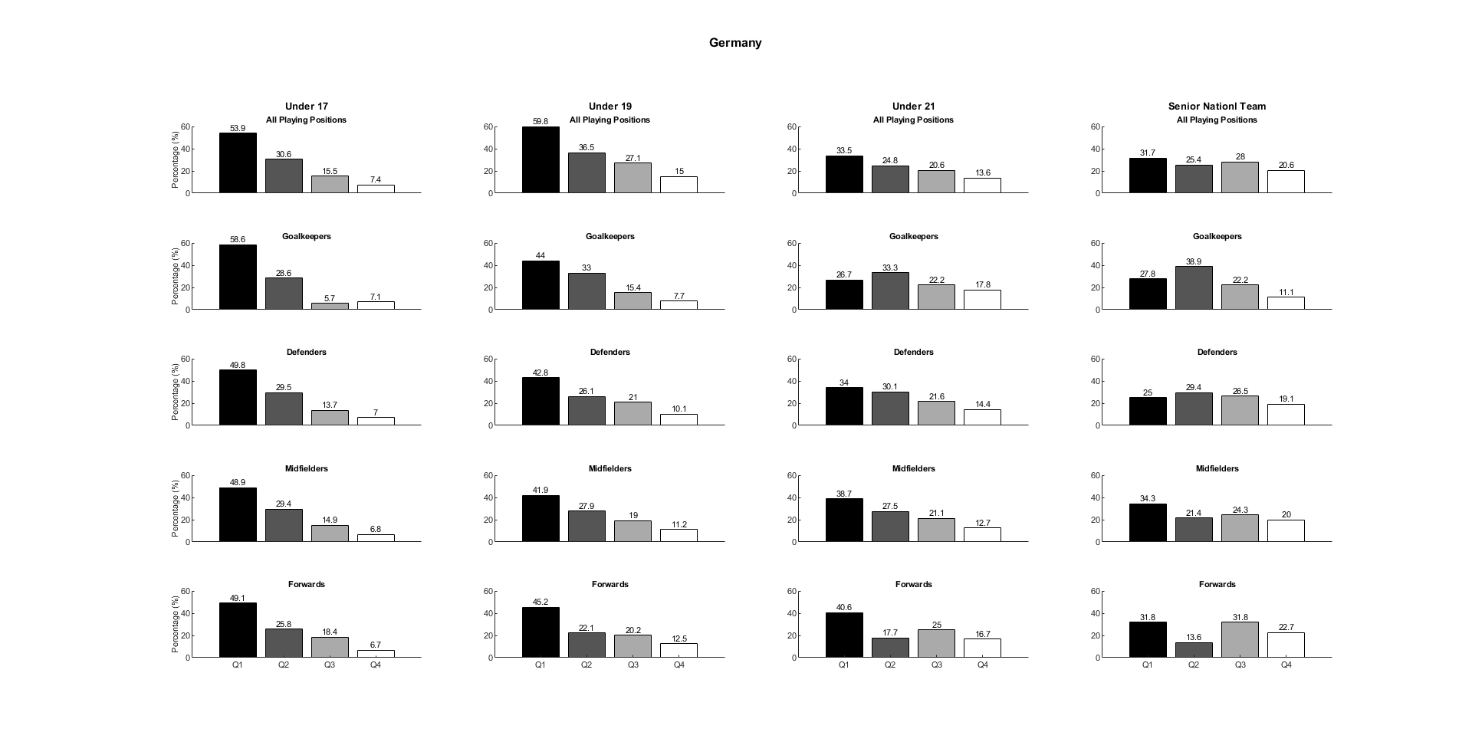
 Legend: Birth quartile percentage distributions presented individually for all playing positions (i.e. goalkeepers, defenders, midfielders and forwards) for U17, U19, U21 and senior German players.


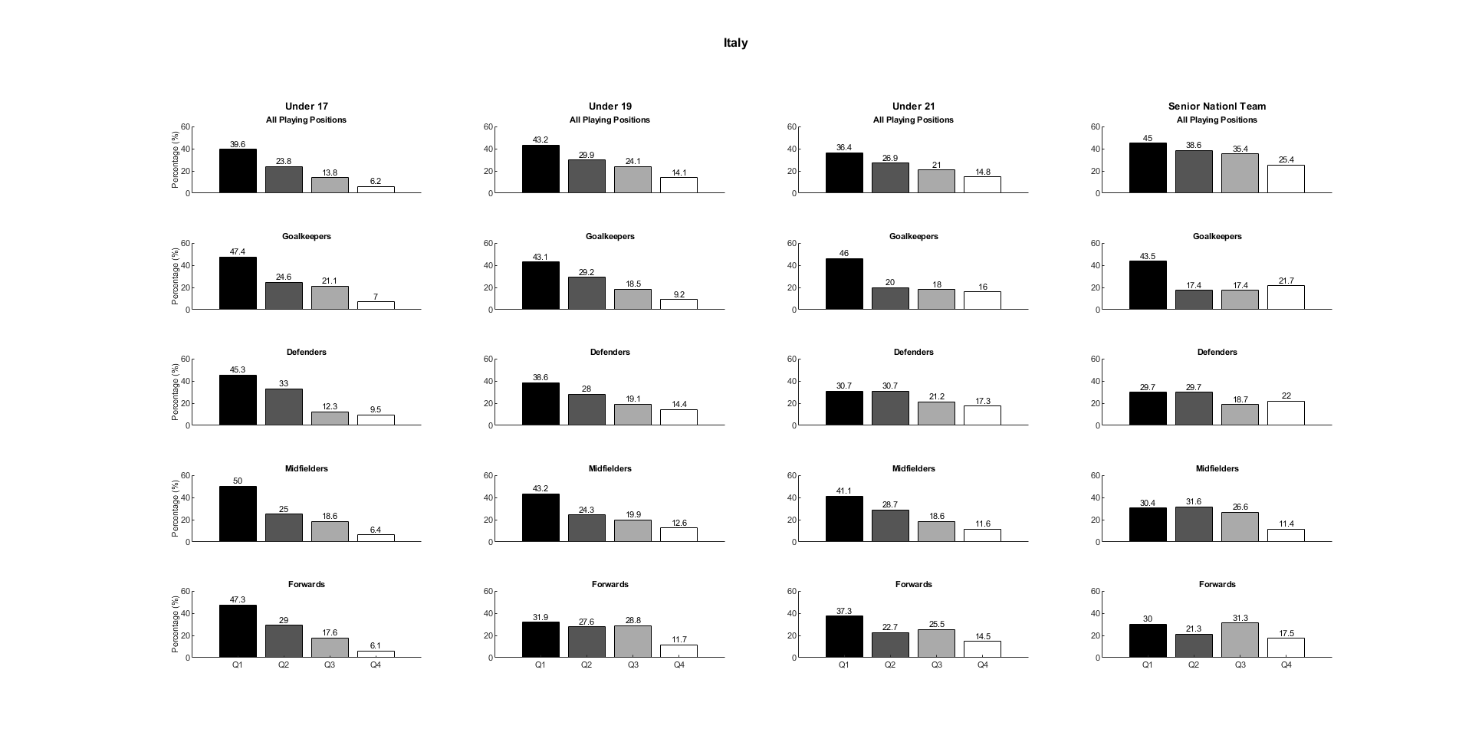


Legend: Birth quartile percentage distributions presented individually for all playing positions (i.e. goalkeepers, defenders, midfielders and forwards) for U17, U19, U21 and senior Italian players.


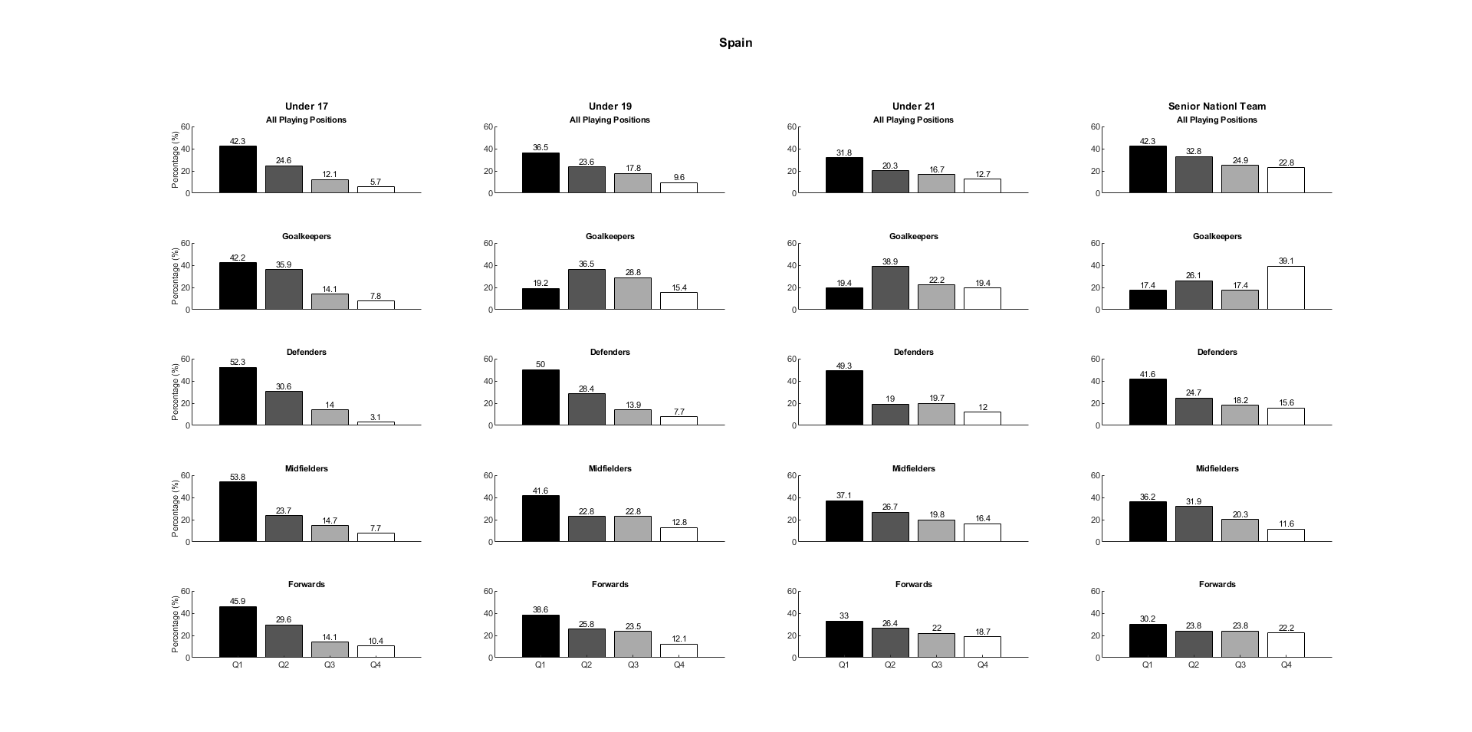


Legend: Birth quartile percentage distributions presented individually for all playing positions (i.e. goalkeepers, defenders, midfielders and forwards) for U17, U19, U21 and senior Spanish players.


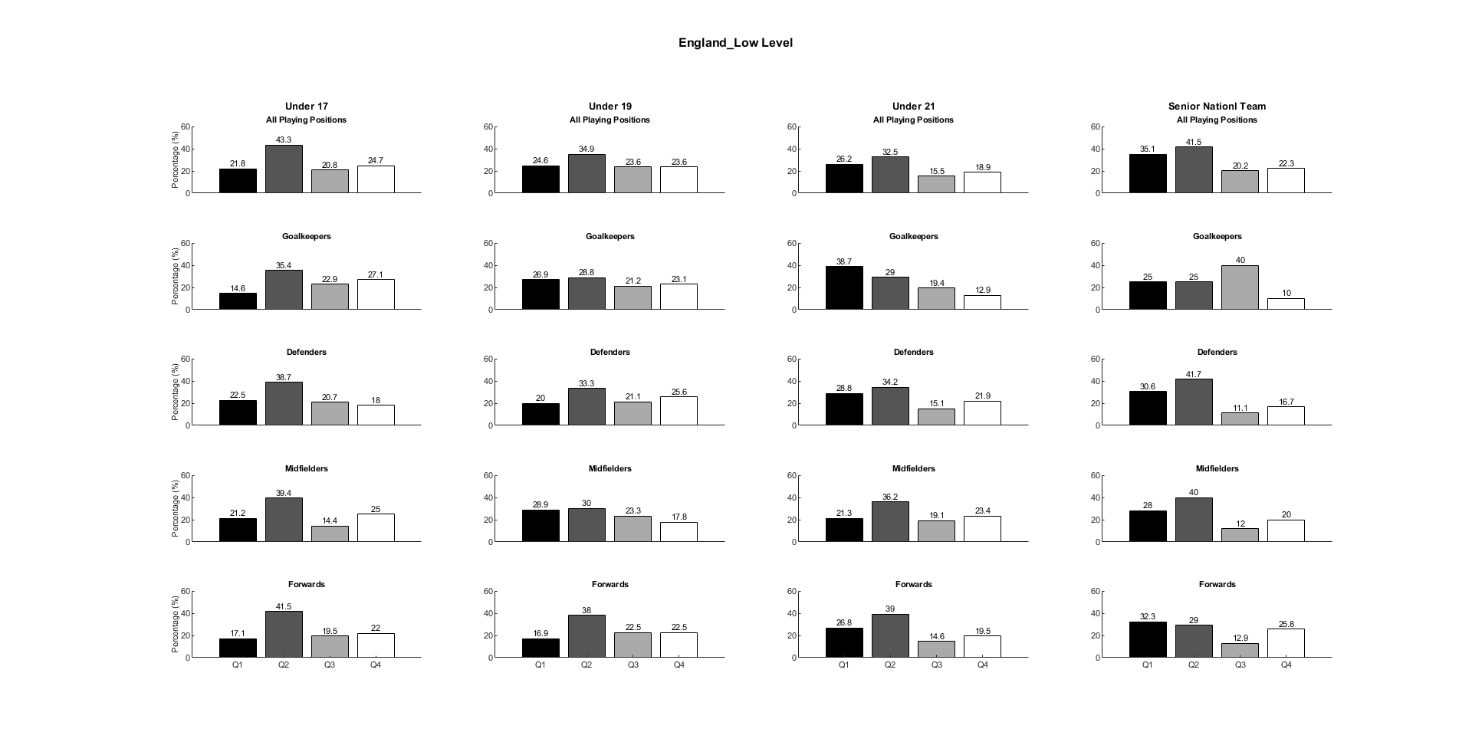

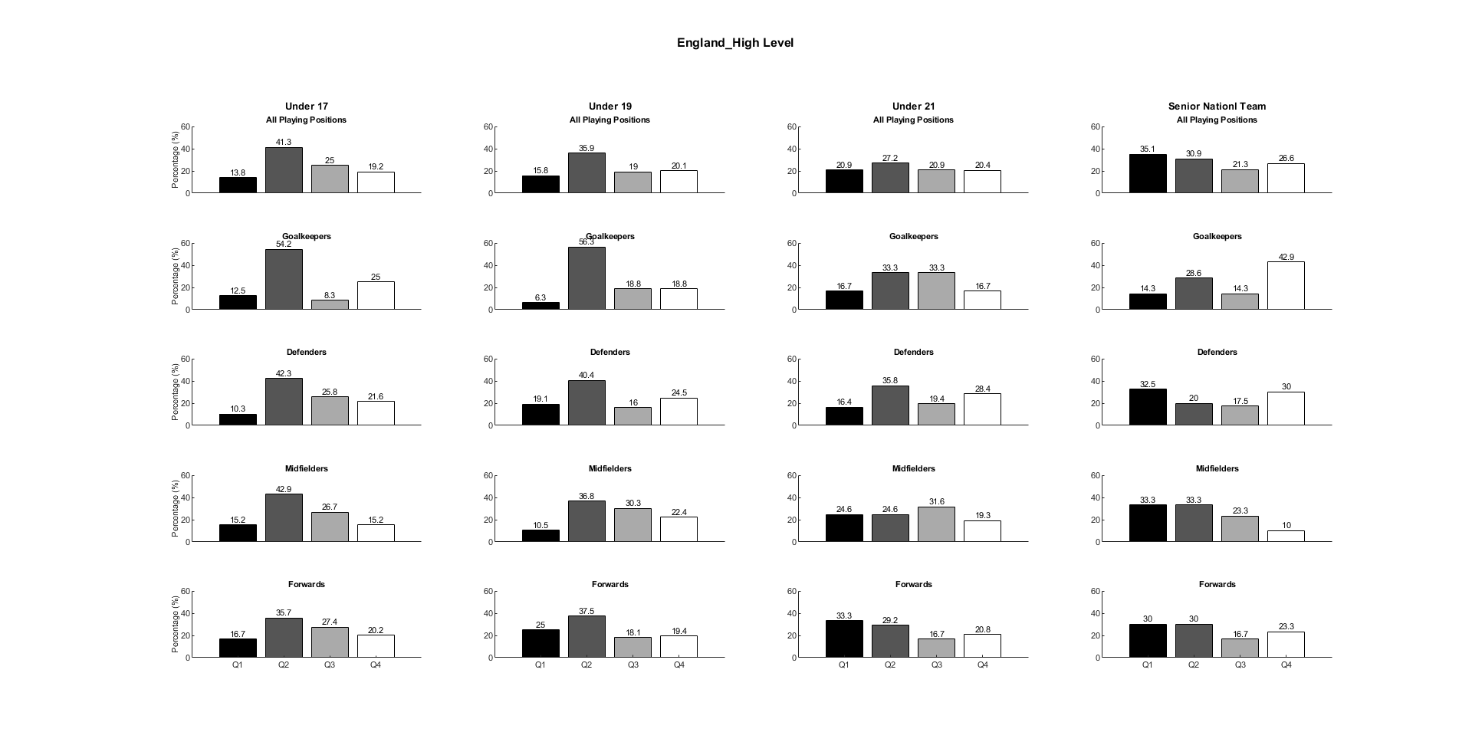
 Legend: Birth quartile percentage distributions presented individually for all playing positions (i.e. goalkeepers, defenders, midfielders and forwards) for English U17, U19, U21 and senior players. The upper panel shows the data for the low competition level category, the lower panel for the high competition level category.


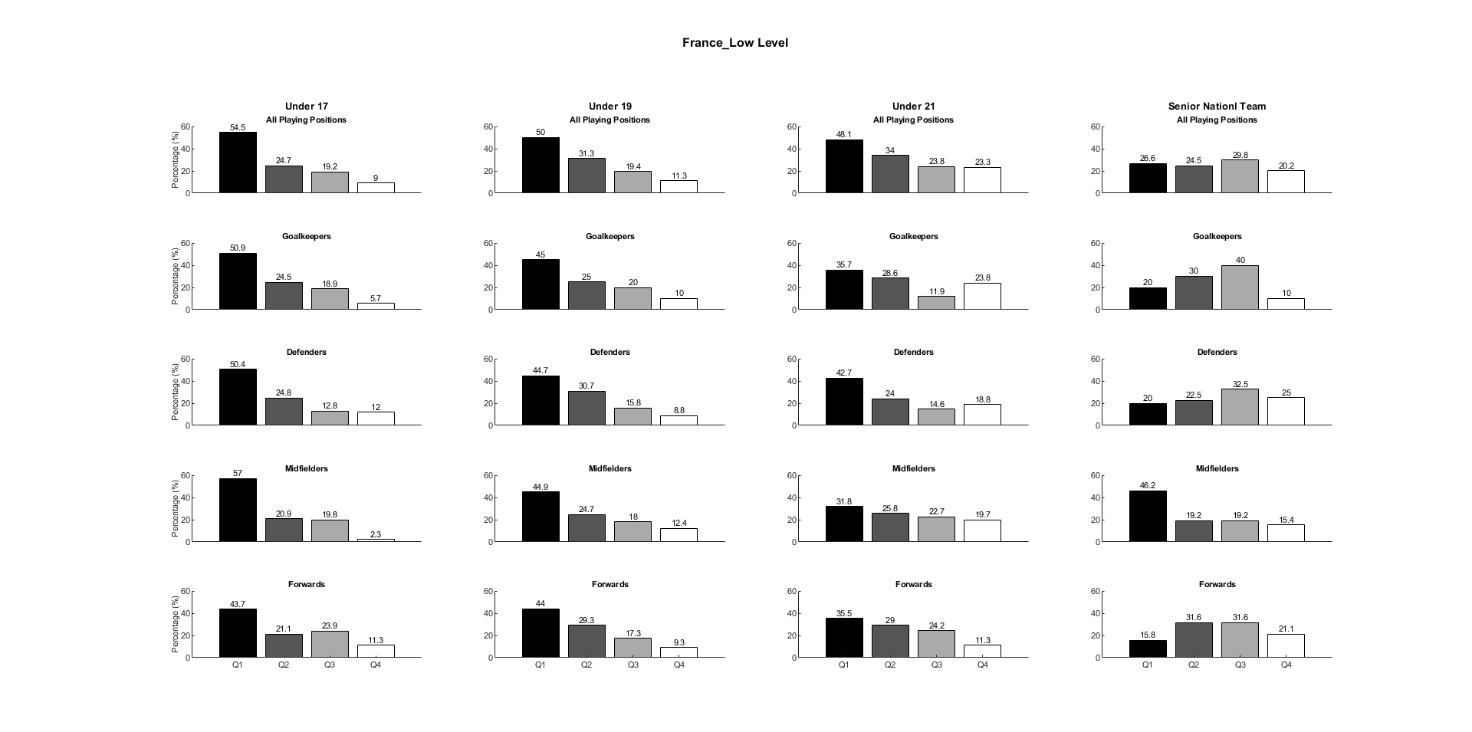

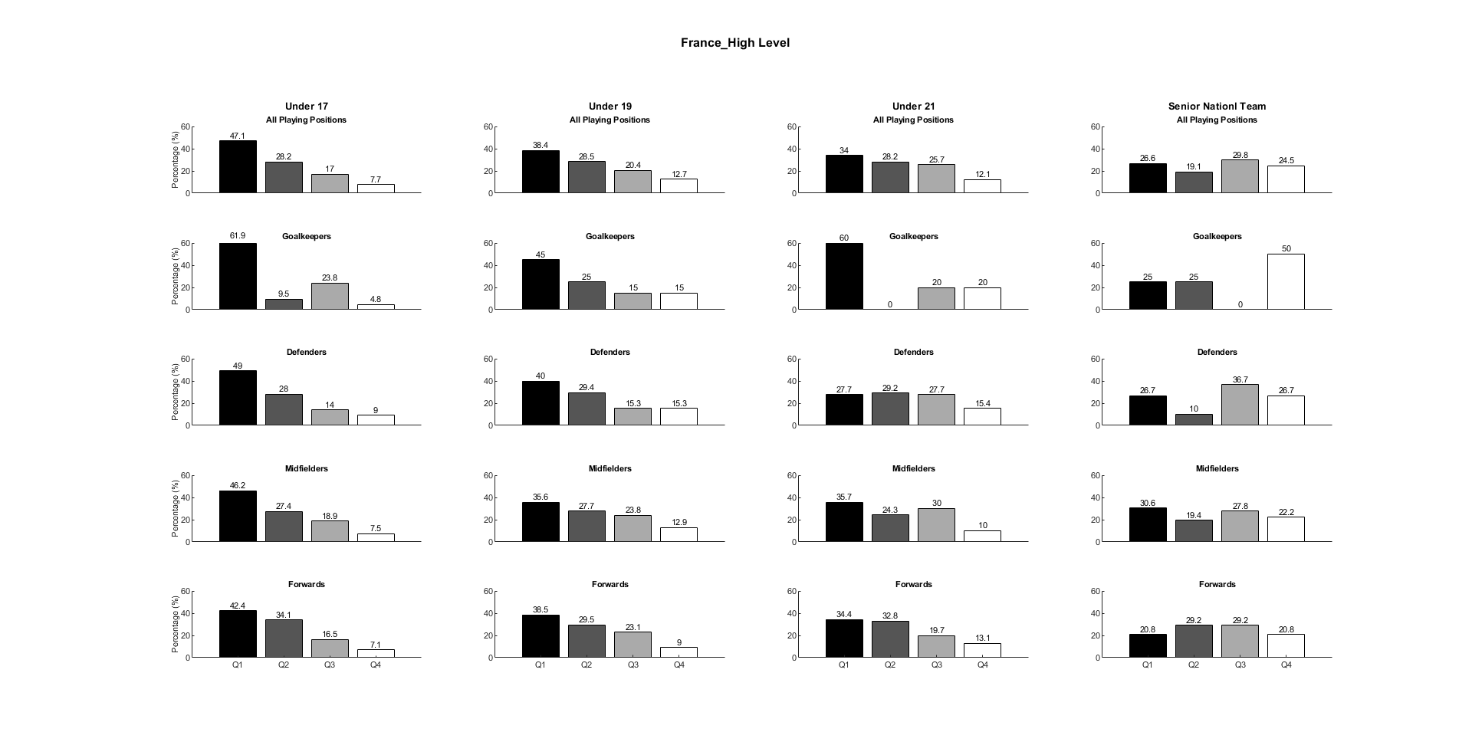
Legend: Birth quartile percentage distributions presented individually for all playing positions (i.e. goalkeepers, defenders, midfielders and forwards) for French U17, U19, U21 and senior players. The upper panel shows the data for the low competition level category, the lower panel for the high competition level category.
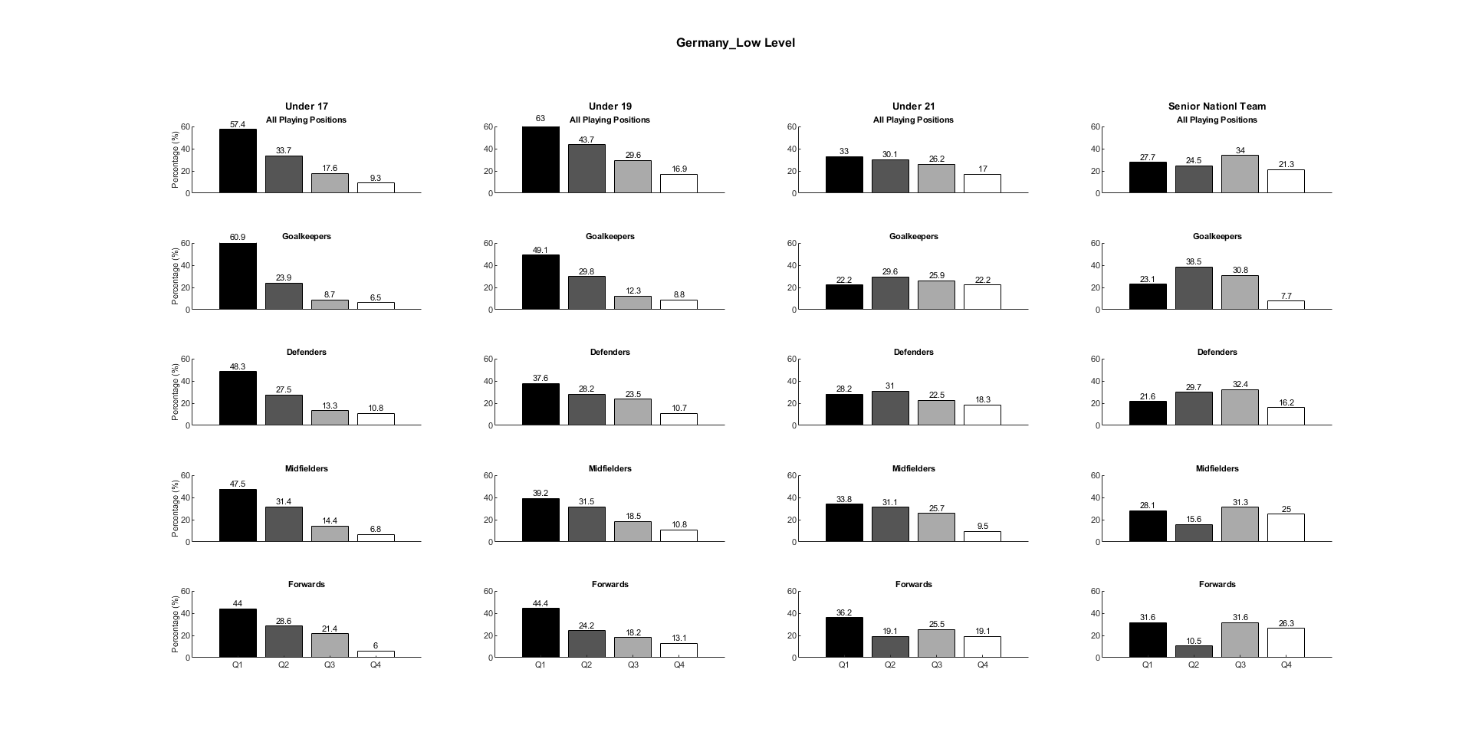

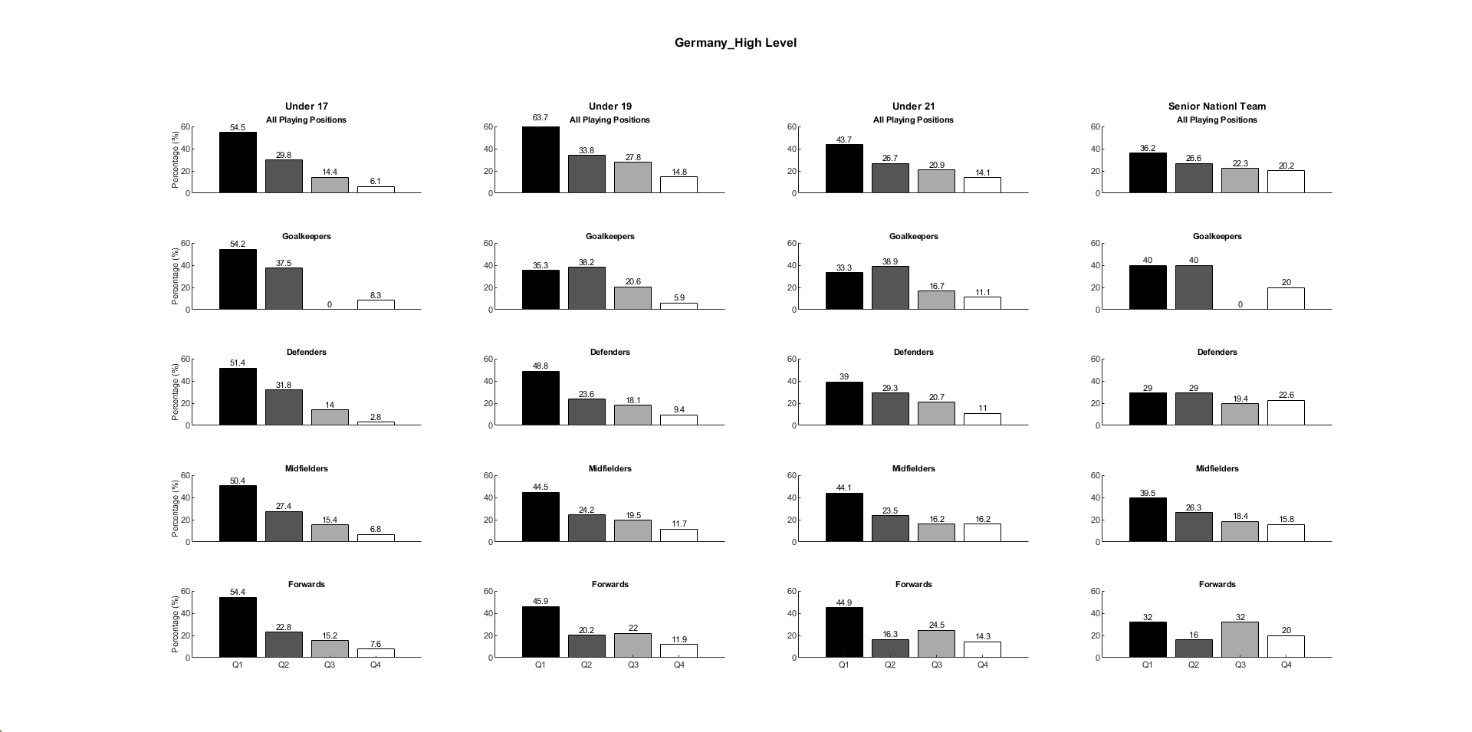
 Legend: Birth quartile percentage distributions presented individually for all playing positions (i.e. goalkeepers, defenders, midfielders and forwards) for German U17, U19, U21 and senior players. The upper panel shows the data for the low competition level category, the lower panel for the high competition level category.
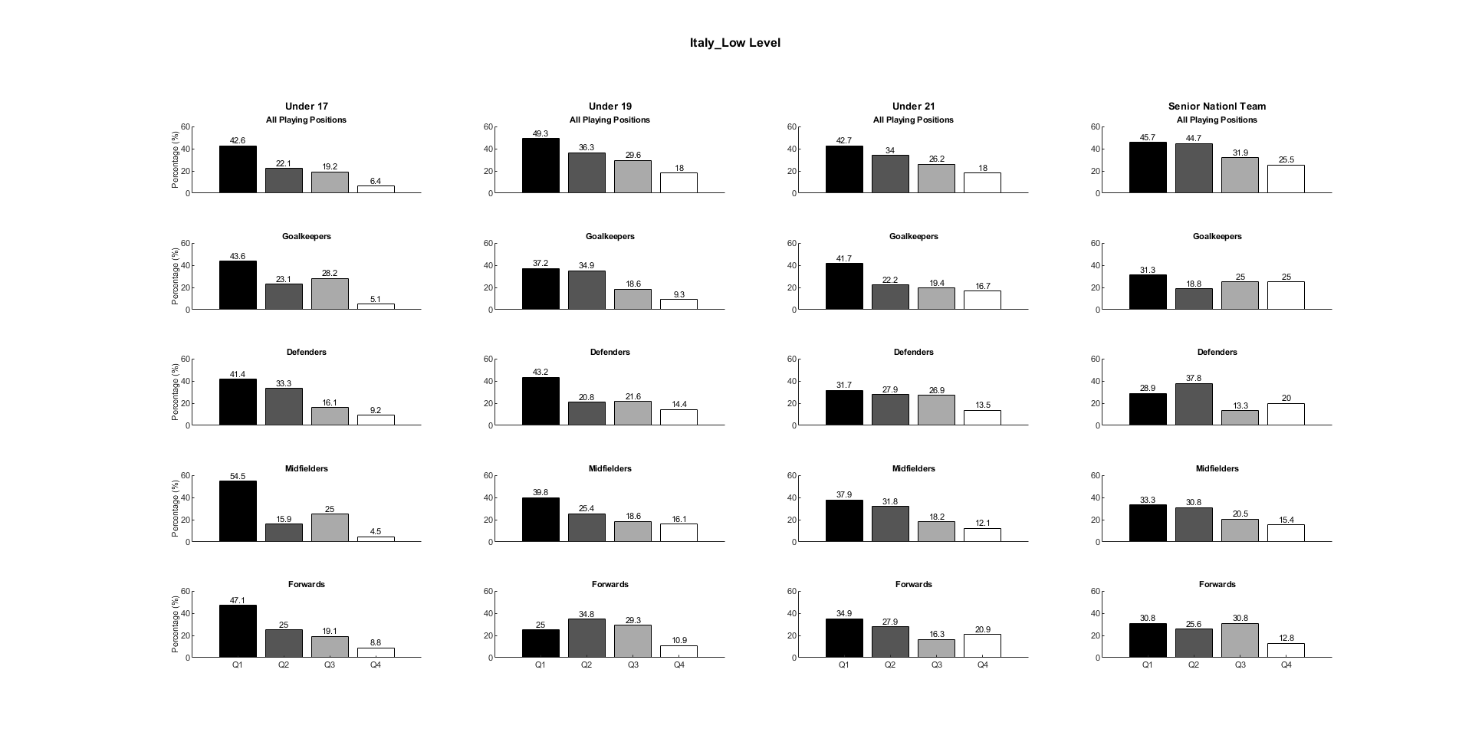

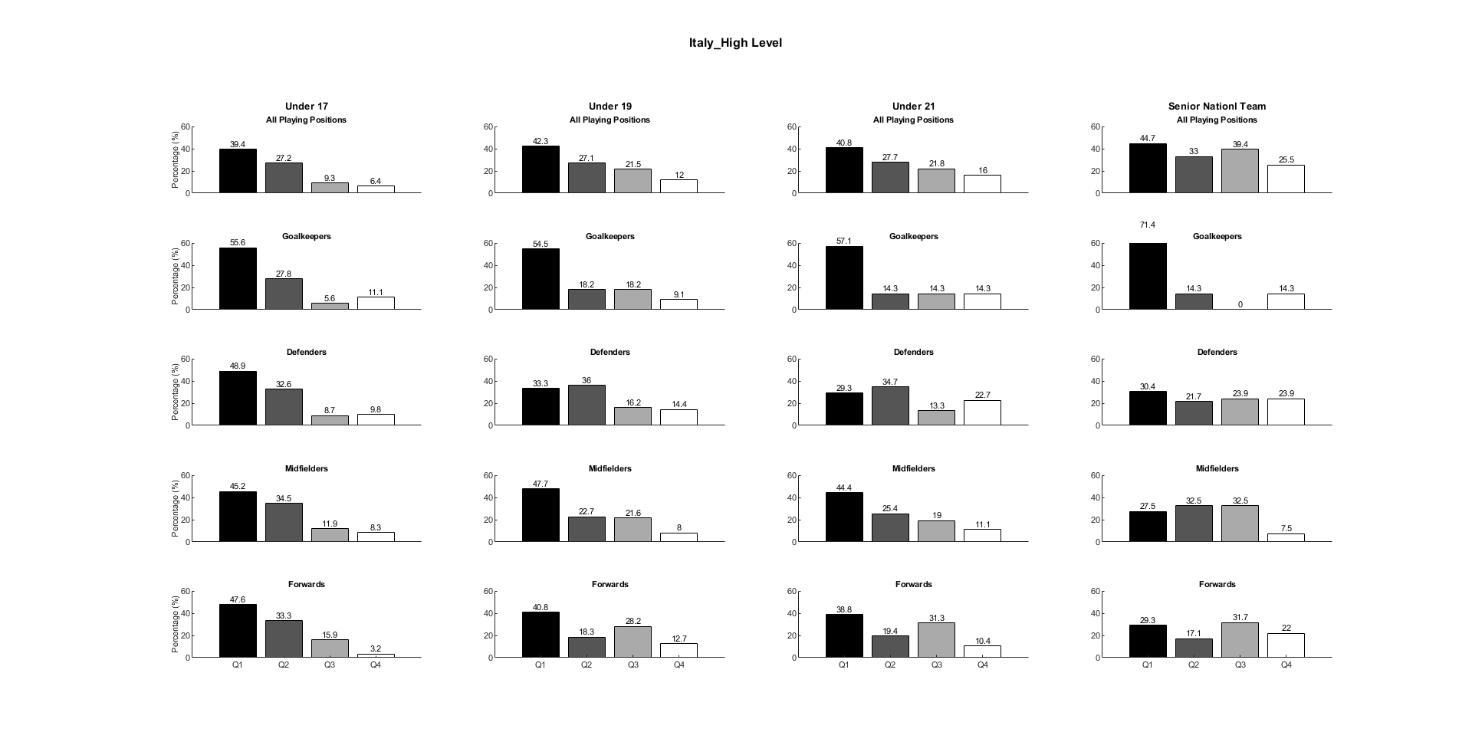
 Legend: Birth quartile percentage distributions presented individually for all playing positions (i.e. goalkeepers, defenders, midfielders and forwards) for Italian U17, U19, U21 and senior players. The upper panel shows the data for the low competition level category, the lower panel for the high competition level category.
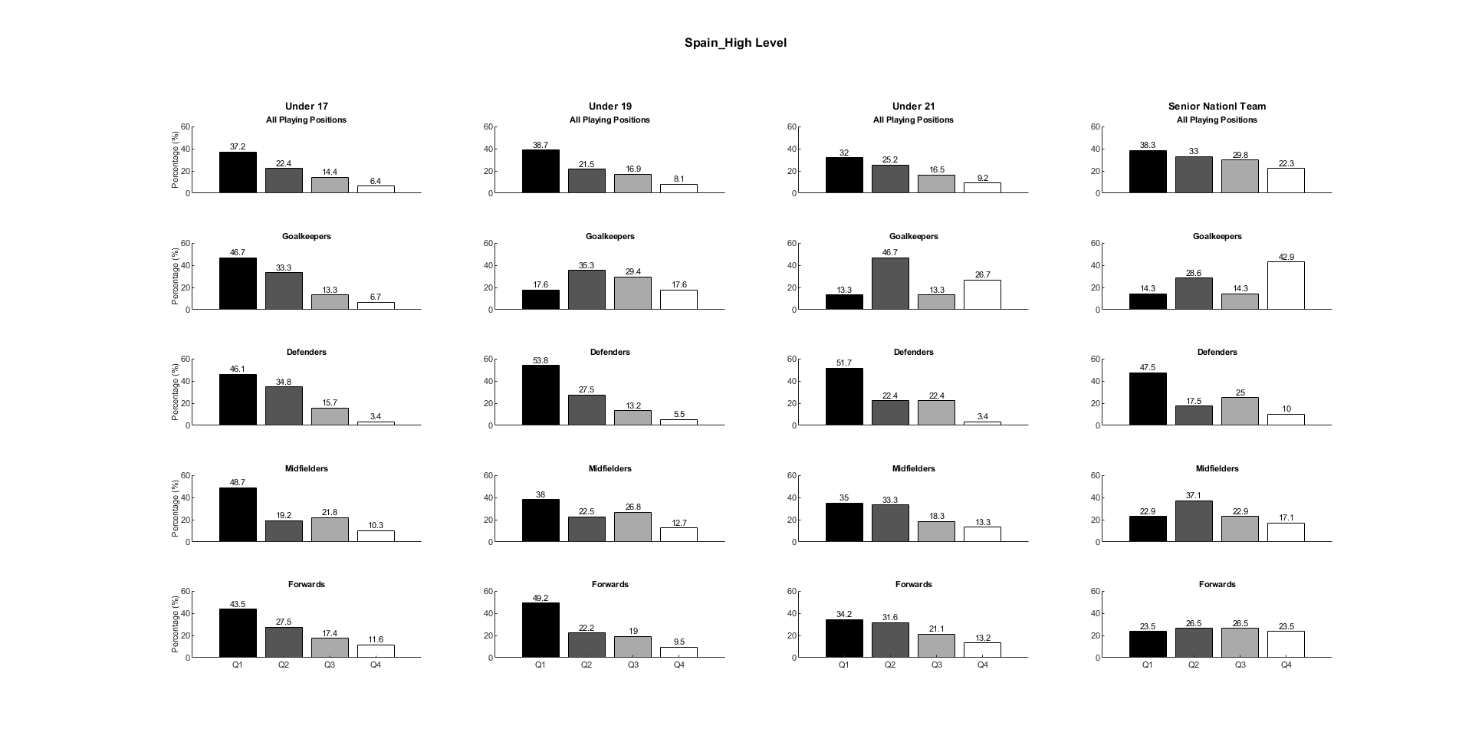

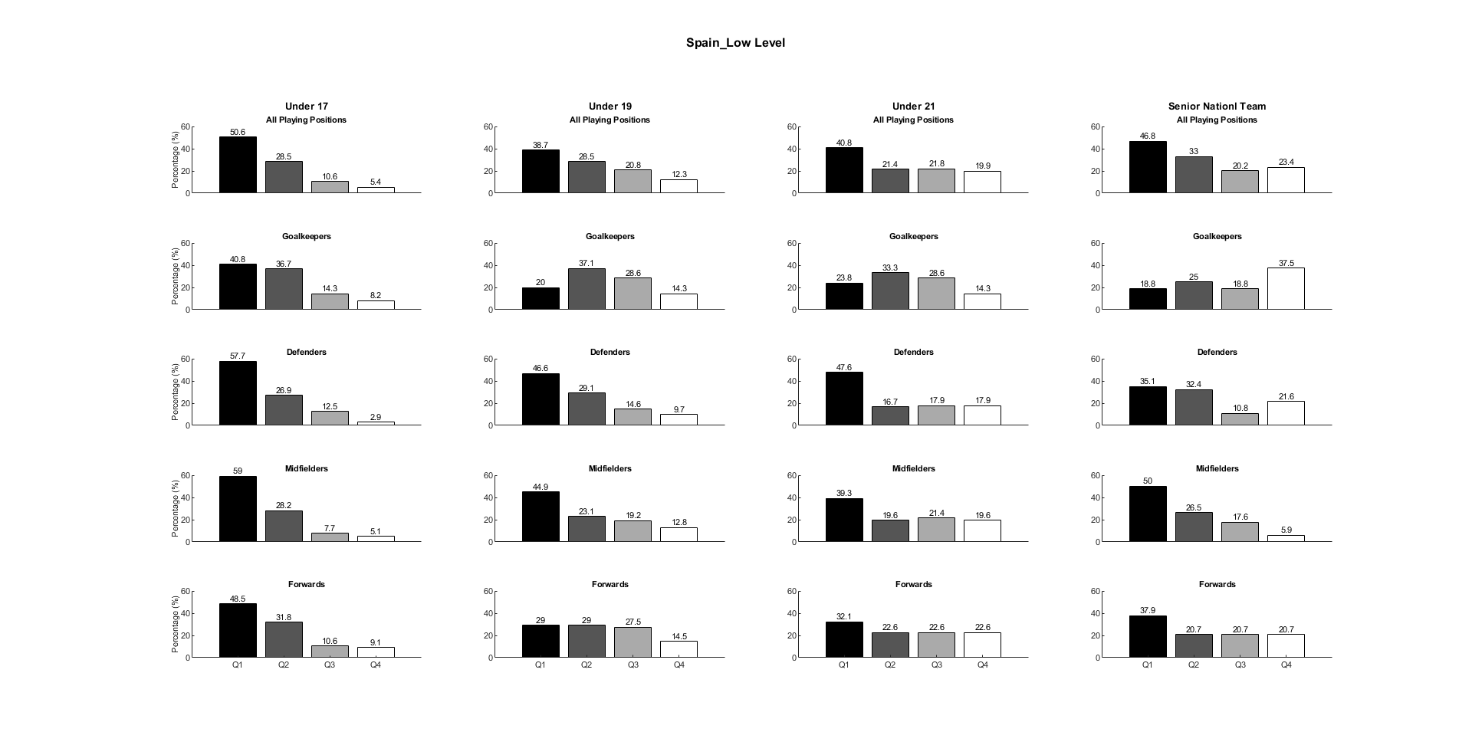


Legend: Birth quartile percentage distributions presented individually for all playing positions (i.e. goalkeepers, defenders, midfielders and forwards) for Spanish U17, U19, U21 and senior players. The upper panel shows the data for the low competition level category, the lower panel for the high competition level category.
