# Supplementary material for: Career trajectories, transition rates, and birthdate distributions: the rocky road from youth to senior level in men's European football
Source: Front Sports Act Living. 2024 Jul 17;6:1420220. doi: 10.3389/fspor.2024.1420220 (PMC11288826; doi:10.3389/fspor.2024.1420220)
Supplement: Supplementary file 2 [file Datasheet2.docx]

| Supplementary 2. Logistical regression outcomes | | | | | | |
| --- | --- | --- | --- | --- | --- | --- |
|  | U17  N=158 | | U19  N=288 | | U21  N=380 | |
|  | B (SE) | OR [95% CI] | B (SE) | OR [95% CI] | B (SE) | OR [95% CI] |
| Q1 Vs Q2 | 0.34 (0.20) ^¥^ | 1.40 [0.95, 2.08] | 0.09 (0.16) | 1.09 [0.79, 1.51] | 0.07 (0.16) | 1.07 [0.78, 1.46] |
| Q1 Vs Q3 | 0.12 (0.26) | 1.13 [0.67, 1.85] | 0.17 (0.18) | 1.19 [0.83, 1.69] | 0.21 (0.17) | 1.24 [0.89, 1.72] |
| Q1 Vs Q4 | 0.68 (0.27)^*^ | 1.98 [1.15, 3.32] | 0.38 (0.19)^*^ | 1.47 [1.00, 2.13] | 0.23 (0.19) | 1.25 [0.87, 1.80] |
| **Notes:** Q1, first quartile; Q2, second quartile; Q3, third quartile; Q4, fourth quartile; B, estimated beta score: SE, Standardized Error; OR, odds ratio and 95% confidence intervals [95% CI]; ^***^, p<0.001; ^**^, p<0.01; ^*^, p<0.05; ^¥^ , near p=0.05. | | | | | | |

| Table . Logistical regression outcomes | | | | | | |
| --- | --- | --- | --- | --- | --- | --- |
|  | U17  N=158 | | U19  N=288 | | U21  N=380 | |
|  | B (SE) | OR [95% CI] | B (SE) | OR [95% CI] | B (SE) | OR [95% CI] |
| Q1 Vs Q2 | 0.34 (0.21) ^¥^ | 1.41 [0.94, 2.12] | 0.05 (0.17) | 1.05 [0.76, 1.45] | 0.03 (0.16) | 1.03 [0.75, 1.41] |
| Q1 Vs Q3 | 0.14 (0.26) | 1.15 [0.68, 1.89] | 0.15 (0.18) | 1.16 [0.81, 1.65] | 0.2 (0.17) | 1.22 [0.87, 1.7] |
| Q1 Vs Q4 | 0.71 (0.28)^*^ | 2.04 [1.16, 3.49] | 0.33 (0.19) ^¥^ | 1.39 [0.95, 2.04] | 0.19 (0.19) | 1.21 [0.84, 1.75] |
| France Vs England | 0.12 (0.28) | 1.13 [0.66, 1.95] | 0.36 (0.21) ^¥^ | 1.44 [0.95, 2.18] | 0.79 (0.2)^***^ | 2.21 [1.49, 3.31] |
| France Vs Italy | 0.05 (0.28) | 1.06 [0.62, 1.82] | -0.12 (0.21) | 0.88 [0.59, 1.33] | 0.59 (0.2)^**^ | 1.81 [1.22, 2.68] |
| France Vs Germany | -0.04 (0.3) | 0.96 [0.53, 1.71] | -0.19 (0.22) | 0.82 [0.54, 1.26] | 0.38 (0.2) ^¥^ | 1.46 [0.99, 2.17] |
| France Vs Spain | 0.5 (0.27) | 1.65 [0.98, 2.82] | 0.49 (0.21)^*^ | 1.63 [1.09, 2.47] | 0.84 (0.2)^***^ | 2.31 [1.56, 3.43] |
| **Notes:** Q1, first quartile; Q2, second quartile; Q3, third quartile; Q4, fourth quartile; B, estimated beta score: SE, Standardized Error; OR, odds ratio and 95% confidence intervals [95% CI]; ^***^, p<0.001; ^**^, p<0.01; ^*^, p<0.05; ^¥^ , near p=0.05. | | | | | | |
